# Experiences and Challenges of Emerging Online Health Services Combating COVID-19 in China: Retrospective, Cross-Sectional Study of Internet Hospitals

**DOI:** 10.2196/37042

**Published:** 2022-06-01

**Authors:** Fangmin Ge, Huan Qian, Jianbo Lei, Yiqi Ni, Qian Li, Song Wang, Kefeng Ding

**Affiliations:** 1 The Second Affiliated Hospital School of Medicine Zhejiang University Hangzhou China; 2 Center for Medical Informatics Health Science Center Peking University Beijing China; 3 School of Management Zhejiang University Hangzhou China

**Keywords:** COVID-19, telehealth, e-consultation, dynamics of health care topics, China health system

## Abstract

**Background:**

Internet-based online virtual health services were originally an important way for the Chinese government to resolve unmet medical service needs due to inadequate medical institutions. Its initial development was not well received. Then, the unexpected COVID-19 pandemic produced a tremendous demand for telehealth in a short time, which stimulated the explosive development of internet hospitals. The Second Affiliated Hospital of Zhejiang University (SAHZU) has taken a leading role in the construction of internet hospitals in China. The pandemic triggered the hospital to develop unique research on health service capacity under strict quarantine policies and to predict long-term trends.

**Objective:**

This study aims to provide policy enlightenment for the construction of internet-based health services to better fight against COVID-19 and to elucidate future directions through an in-depth analysis of 2 years of online health service data gleaned from SAHZU’s experiences and lessons learned.

**Methods:**

We collected data from SAHZU Internet Hospital from November 1, 2019, to September 16, 2021. Data from over 900,000 users were analyzed with respect to demographic characteristics, demands placed on departments by user needs, new registrations, and consultation behaviors. Interrupted time series (ITS) analysis was adopted to evaluate the impact of this momentous emergency event and its long-term trends. With theme analysis and a defined 2D model, 3 investigations were conducted synchronously to determine users’ authentic demands on online hospitals.

**Results:**

The general profile of internet hospital users is young or middle-aged women who live in Zhejiang and surrounding provinces. The ITS model indicated that, after the intervention (the strict quarantine policies) was implemented during the outbreak, the number of internet hospital users significantly increased (β_2=105.736, *P*<.001). Further, long-term waves of COVID-19 led to an increasing number of users following the outbreak (β_3=0.167, *P*<.001). In theme analysis, we summarized 8 major demands by users of the SAHZU internet hospital during the national shutdown period and afterwards. Online consultations and information services were persistent and universal demands, followed by concerns about medical safety and quality, time, and cost. Users’ medical behavior patterns changed from onsite to online as internet hospital demands increased.

**Conclusions:**

The pandemic has spawned the explosive growth of telehealth; as a public tertiary internet hospital, the SAHZU internet hospital is partially and irreversibly integrated into the traditional medical system. As we shared the practical examples of 1 public internet hospital in China, we put forward suggestions about the future direction of telehealth. Vital experience in the construction of internet hospitals was provided in the normalization of COVID-19 prevention and control, which can be demonstrated as a model of internet hospital management practice for other medical institutions.

## Introduction

Digital health care is on the frontline in the fight against COVID-19, during which strict quarantine policy deterred most public access to onsite health care [1]. In response, online services from internet hospitals were quickly mobilized and dramatically upgraded as emergency relief measures for COVID-19 prevention and control in China. The pandemic accelerated the development of telehealth, including complementary services to existing departments, policy support, and rapid development and implementation of internet hospitals [2]. Internet hospitals, in general, are online medical platforms that combine online and offline access to medical institutions to provide a variety of telehealth services directly to patients. To date, 4 kinds of services have been equipped, including convenience services (booking appointments, checking test results), online medical services (electronic prescriptions), telemedicine (health education), and related support (follow-up consultations) [3]. There are 3 types of internet hospitals in China: government-oriented, hospital-oriented, and enterprise-oriented [4]. Routinely, Chinese patients go to hospitals, repeatedly queuing for registration, inquiries, or medical checkups in different departments [5]. Internet hospitals are able to render multiple services and offer essential medical support, surmounting geographical and time-related barriers [6]. Generally, internet hospitals in huge demand alleviate the imbalance of limited medical resources and increased burden of chronic diseases [7].

In China, the internet hospital is part of an ambitious plan, “Healthy China 2030 initiative,” released by the Chinese government in 2016. One of the important tenants of the plan is to make full use of internet technology to promote the integration of the internet and medical care, which is known as the “internet-plus-healthcare plan.” The construction of internet hospitals is an important part of the plan. The outbreak of COVID-19 greatly stimulated the explosive growth of internet hospitals. In 2016, there were only 25 internet hospitals [8]. By December 31, 2020, 1004 [9] had been established, and by June 2021, this number had increased to 1600 [10]. By 2017, the market size of internet medical services in China will be 32.5 billion yuan, with an estimated 250 million users [11]. The rapid excessive growth of internet hospitals urgently calls for a summary of relevant experience and construction guidance to ensure the healthy development of internet hospitals.

Worldwide, studies verified that, in the initial stage of the pandemic, there was a considerable amount of emerging literature on telehealth in most high-income countries [11]. For example, in the United States, approximately 60.0% of health care consumers reported that they first search online for information about an intended doctor through the internet hospital site or physician-rating websites before making a choice [12]. In addition, 59% of health care consumers confirm their choice based on the evaluation of doctors by internet hospitals or physician-rating websites [13]. However, the application of telehealth in resource-limited settings and low- and middle-income countries must be established to make the most of its potential and transform health care for the world’s population [14]. The definition, functions, boundaries, and hidden problems of internet hospitals are in urgent need of updated consensus to realize their potential and promote health care in the future.

SAHZU has played a pioneering and leading role in China's development of internet hospitals. SAHZU is located in Hangzhou, the cradle of well-known internet enterprises (eg, Alibaba and NetEase). SAHZU is one of the oldest public general hospitals, ranking in the top 10 among national general hospitals in China. As the “forerunner” of online medical care in China, the hospital took the lead in 2017 to launch an internet hospital and has continuously updated its capabilities. To date, almost 900,000 users have registered at SAHZU's Internet Hospital, which provides services to more than 1 million individuals per month. It initiated free online consultation and medication delivery services from January 27, 2020, to March 27, 2020, covering the lockdown period of the province. On March 15, 2020, China's first relevant group standard, Regulations on Online Consultation Services for Infectious Diseases, was released [15]. In the following 18 months, the hospital modified its strategies in promoting smart-assisted services as COVID-19 surged and subsided.

The pandemic triggered this unique research on health service capacity under strict quarantine policies. Only a few studies have reported the maximum usage of online hospital services during major public health emergencies. The strength of this study might be long-term follow-ups that further identify users’ behavior patterns and provide implications for the construction of global internet hospitals in the COVID-19 era. Of note, we captured the authentic demands of online health care seekers during the national blockade at the beginning of the pandemic. This study aimed to demonstrate the changes and trends in internet hospital applications during the COVID-19 pandemic in China.

## Methods

### Study Timeline

**Figure 1:**
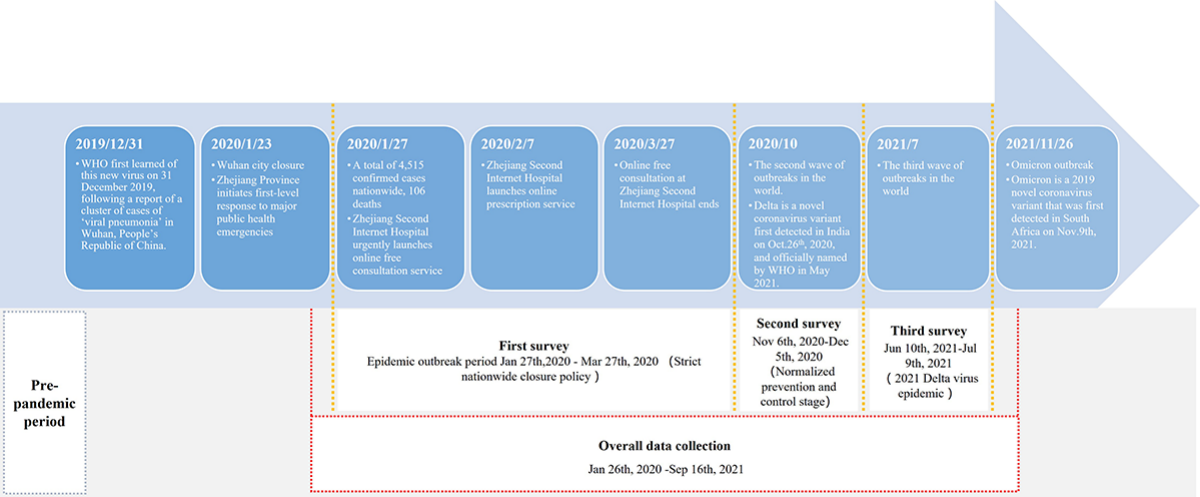
Study timeline. WHO: World Health Organization.

On January 30, 2020, the World Health Organization declared COVID-19 to be a public health emergency of international concern. SAHZU efficiently responded by establishing a free online consultation portal and received more than 10,000 consultation requests in the following 2 months. On February 7, 2020, a special online pharmacy was created to circumvent difficulties in obtaining regular medications. As an observational, cross-sectional study, we defined time periods as shown in Figure 1, whereas the investigation timeline primarily synchronized with national policies.

### Data Collection and Analysis Methods

**Figure 2:**
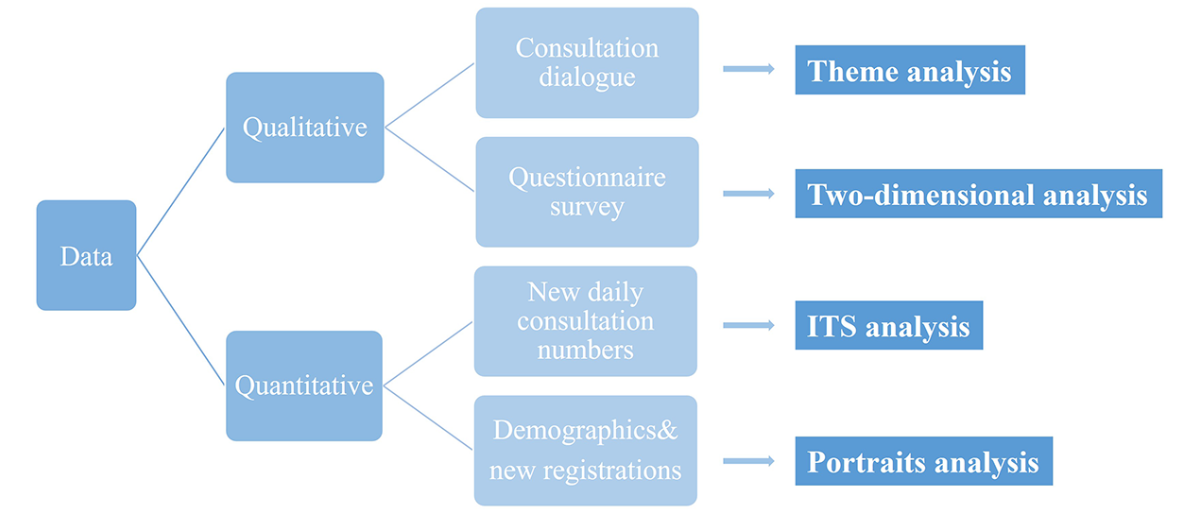
Data sources and analysis methods. ITS: intermittent time series.

The data sources in this paper were divided into 2 categories: quantitative data and qualitative data. Multiple data sources and research methods are illustrated in Figure 2.

#### Data Collection

##### Quantitative Data

Data from all internet hospital users, including daily registration numbers and user demographics (gender, age, geolocation, choice of specific department, and service needs), were collected on August 15, 2021.

##### Qualitative Data

As the basis of the initial research (first survey) and the qualitative research of this study, 15,990 conversation flows during the outbreak period under the national shutdown were reviewed in this study.

The first step was data reduction. This study is based on real-world data, and dialogue between doctors and patients contains grammar errors, dialects, and nonstandard expressions. Thus, the dialogue flow needs to be manually analyzed to address one of the major demands we classified.

Second, there was required prework before the analysis. For instance, more than one kind of demand could be included in a consultation case; therefore, the sum of the counts is not equal to the total number of cases. Moreover, a single user could have initiated several consultation cases with different needs or different patients because the platform allows users to initiate consultations for themselves or their family members using valid patient IDs.

Third, every entry in the data set was de-identified, including the gender, age, patient’s chief complaint and previous diagnoses, the department of the clinician the patient consulted, and the content of the conversation without private information.

Thus, we preprocessed the dialogue between doctors and patients before formally processing the data.

Following the data reduction task, our theme analyses followed 3 steps: (1) theme formation, (2) theme matching along themes and patterns observed in the conversations, and (3) theme comparison across practice sites. Finally, we identified 8 major categories of demands from users, as listed in Table 1.

**Table 1:** Major categories of demands under the national shutdown period.

Number	Major categories of demands
1	Follow-up consultation
2	Drug refill
3	Consultation on common symptoms
4	Consultation on suspected symptoms of COVID-19
5	Information service
6	Psychological support
7	Rescheduling of treatment
8	Guidance on protective measures

The data were independently reviewed by 8 members of the investigative team, making methodological memos, theoretical memos, and preliminary interpretations. Individual researcher analyses and interpretations were discussed by the research team throughout the project. The themes and patterns were further refined, and new themes were cogenerated. All themes were developed through a process of articulating a unifying idea that represented interpretations from multiple data points. Conceptual labels were assigned to organize themes according to a common thread among ideas. In each step, themes were refined, whereby similarly labelled ideas were combined into themes and given more general labels. Disagreements were resolved through group discussion until consensus was reached. Finally, unstructured data were assigned to 8 major categories and then cleaned by the same researcher to keep the classification results unified. In our following question surveys, the users of our sample survey came from the users of our theme analysis.

To further investigate the needs and trends in patient behavior during online health service, we conducted 2 follow-up questionnaire surveys in November 2020 and June 2021, corresponding to the start and end of the second wave of the pandemic, respectively. The questionnaire (see Multimedia Appendix 1) includes 3 questions with fixed options: main reasons for using internet hospitals during the pandemic, changes in their way of accessing medical care after the pandemic, and major concerns about telehealth services. Interviewees were randomly selected from those who used internet services during the pandemic outbreak. At the start and finish of this second wave, 2 random online or telephone surveys were completed. Among the randomly selected 1100 actual internet hospital users for each survey, 1060 and 805 valid questionnaires were collected, respectively. All surveys were conducted anonymously, and each person only answered 3 questions from the predesigned questionnaire.

##### Ethics Approval

Our study protocol and procedures for informed consent before the formal survey were approved by the hospital ethics committee (Approval Number 2021-0761). Participants were required to answer a yes or no question to confirm their willingness to participate voluntarily. After confirmation of the question, the participant was directed to complete the questionnaire.

#### Analytical Methods

##### Interrupted Time Series Analysis

Interrupted time series (ITS) analysis was adopted to evaluate the impact of momentous emergency events (Multimedia Appendix 2). To identify use prompted by the pandemic, we assessed differences in new daily consultation numbers from July 15, 2019, to September 15, 2021, with a baseline period (November 1, 2019, to January 26, 2020) and after the national lockdown response (March 18, 2020, to June 30, 2020). We conducted a multistage comparison of the development of online medical treatment (October 1, 2020, to April 10, 2021, and April 11, 2021, to September 16, 2021); thus, the long-term impact of the pandemic was also considered in the ITS analysis.

##### 2D Analysis

**Figure 3:**
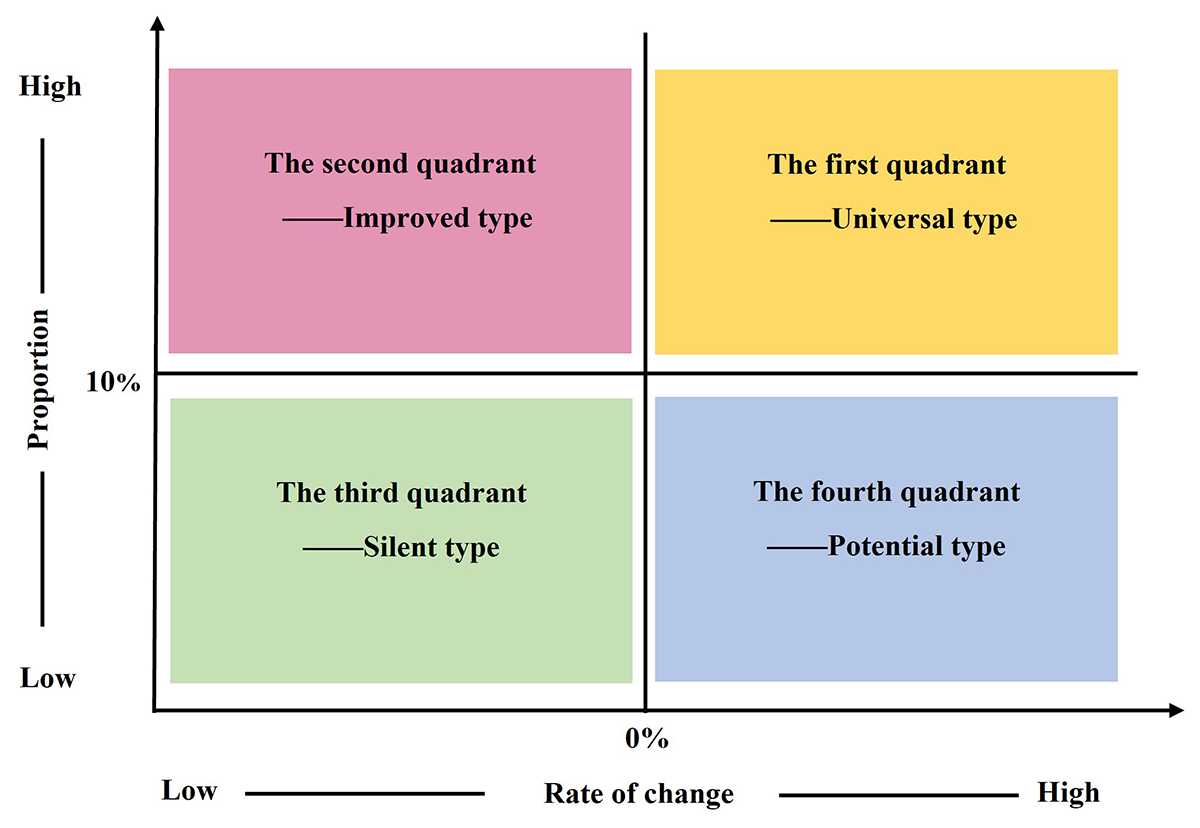
2D model.

To gain deep insight into the changes in patient demands, we modeled the proportion and increase or decrease of each demand through a 2D model. A matrix chart with 4 quadrants was designed; the abscissa is the rate of change, and the ordinate is the distribution. Different functions fall into typical quadrants, as expressed in Figure 3.

The first quadrant is a universal type, with a large proportion of demand and upwards change in different periods. The second quadrant is an improved choice, relatively stable, with a large proportion of demand but downward changes. The third quadrant is a silent type, with a small proportion of demand and downward changes in different periods. The fourth quadrant is the potential needs; the proportion of demand is small, but the changes are upwards. Furthermore, this 2D model was also used to classify the data on changes in patients’ ways of accessing medical care and concerns.

##### Statistical Analysis and Visualization

SPSS 25.0 software (28.0.1; IBM Corp, Armonk, NY) was used for data analysis. Frequencies and percentages were used for categorical data. The chi-square test was adopted for comparisons between groups. A 2-tailed *P* value <.05 was considered statistically significant.

## Results

### Demographic Composition of All Users and Their Online Choices

**Figure 4:**
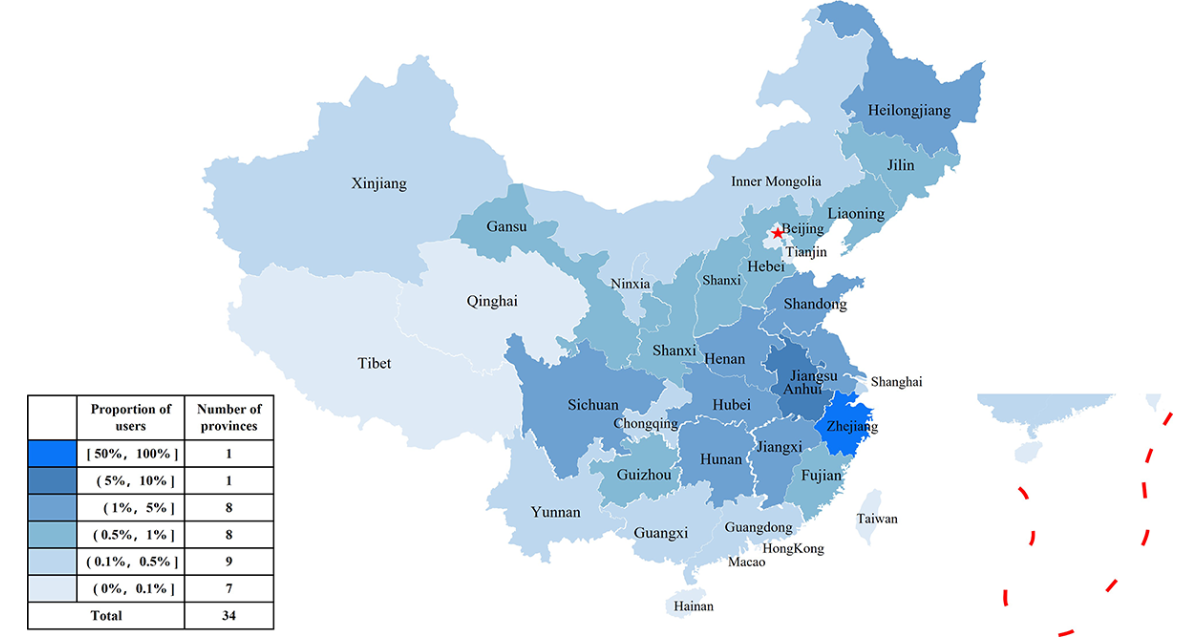
Regional composition of all Second Affiliated Hospital of Zhejiang University School of Medicine (SAHZU) internet hospital users before August 15, 2021.

**Figure 5:**
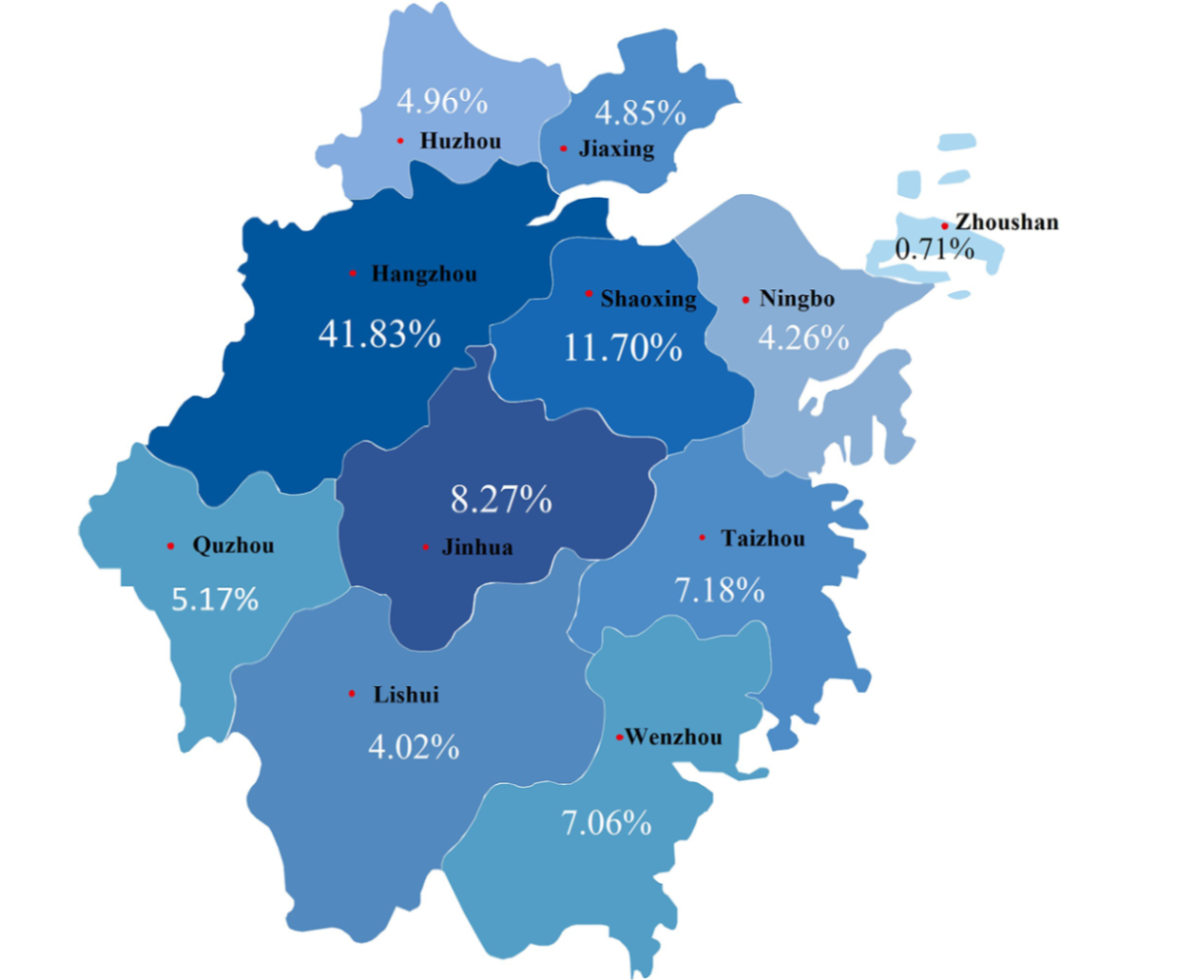
Regional composition of Second Affiliated Hospital of Zhejiang University School of Medicine (SAHZU) internet hospital users in Zhejiang Province before August 15, 2021.

**Figure 6:**
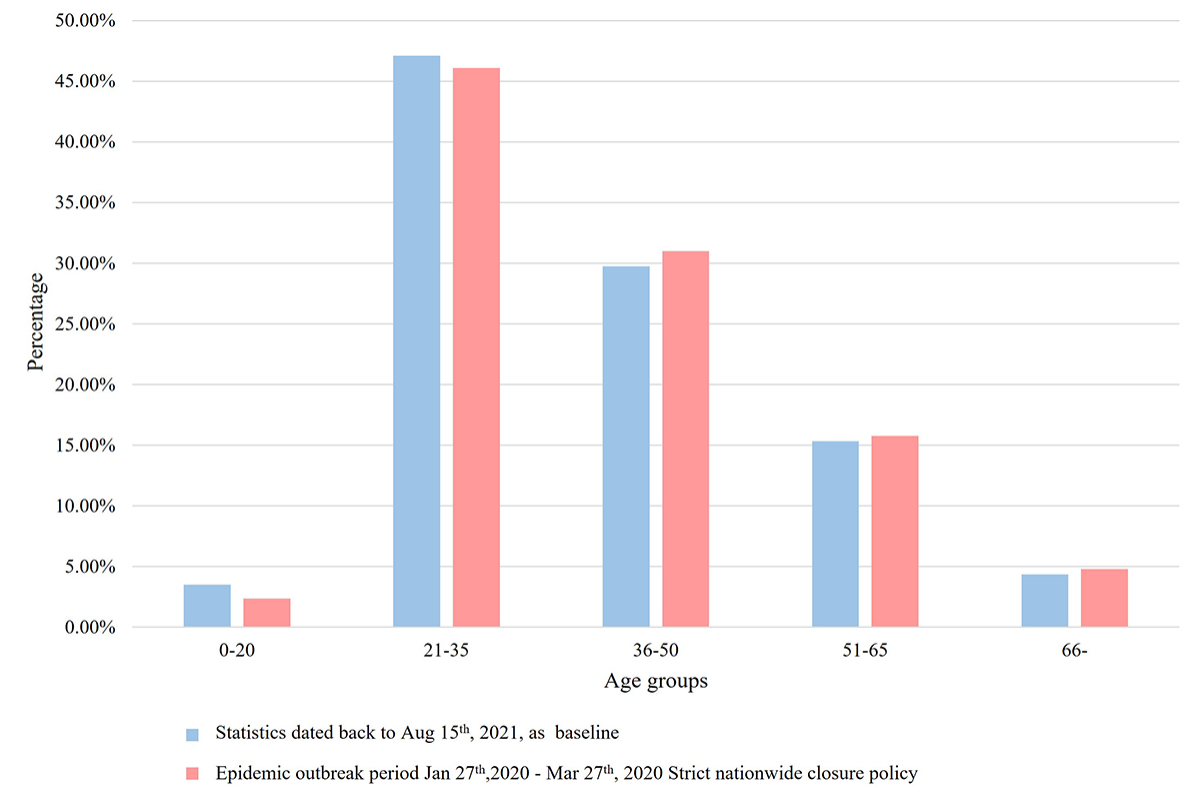
Comparison of the age group composition of Second Affiliated Hospital of Zhejiang University School of Medicine (SAHZU) internet hospital users before August 15, 2021, and those during the pandemic outbreak period from January 27, 2020, to March 27, 2020.

**Figure 7:**
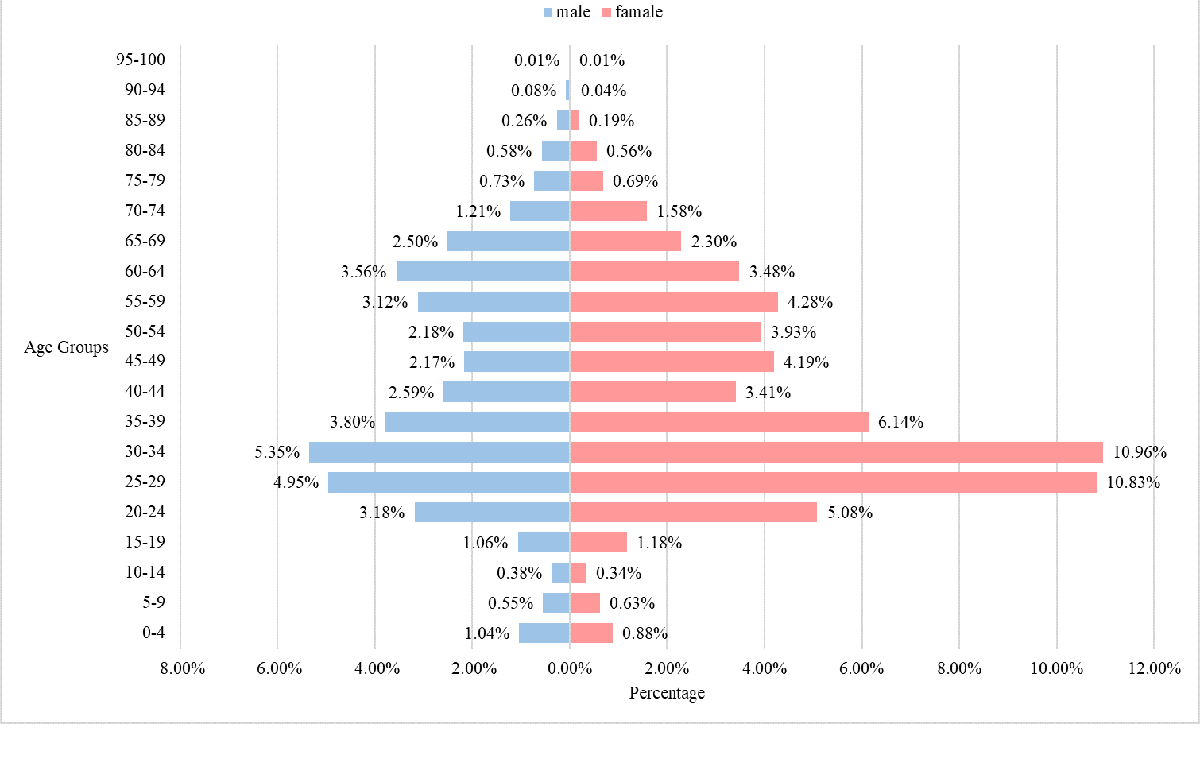
Age and gender distributions for all online consultation participants during the pandemic outbreak period (January 27, 2020, to March 27, 2020). Information for cases such as infants or older adults was apparently completed by their family members.

The demographic composition of internet hospital service users was analyzed based on August 15, 2021 (Figures 4 and 5). In comparison, we also describe the demographic characteristics of online patients from January 27, 2020, to March 27, 2020 (Figure 6) and those active in online consultation during quarantine (Figure 7).

**Figure 8:**
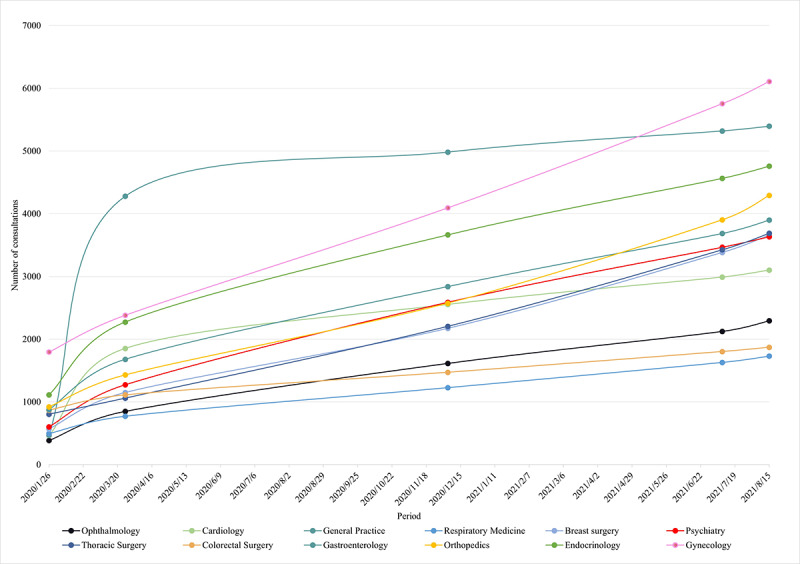
The clinical departments that received the most consultation requests.

**Figure 9:**
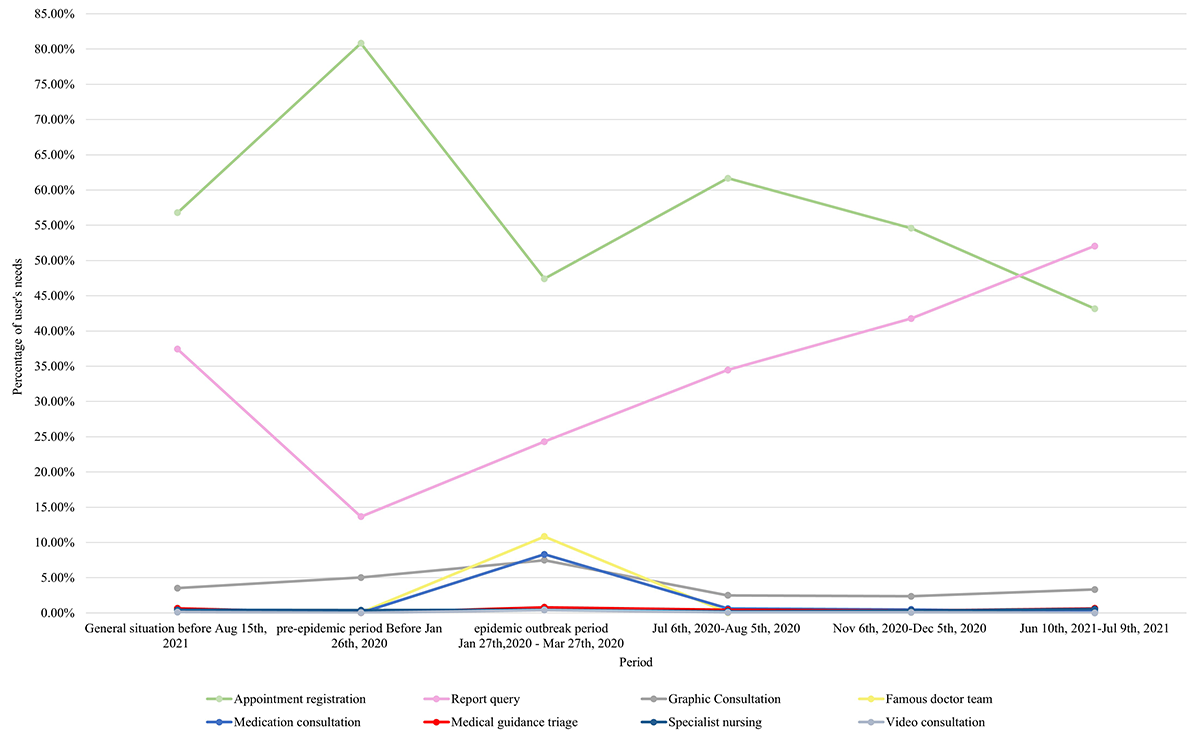
Changes in Second Affiliated Hospital of Zhejiang University School of Medicine (SAHZU) internet hospital users' demands over time.

We illustrate a change in department choice among requests for online consultation (Figure 8), mainly an increase for the gynecology, endocrinology, obstetrics, and orthopedics departments. During the COVID-19 outbreak, the SAHZU internet hospital began to offer free online consultations and assembled a highly responsive team that remained stable afterwards. With respect to the departments focused on the most common and chronic diseases, the number of online consultations increased significantly over the long term. The choice of internet hospital functions (Figure 9) showed that users were mainly interested in appointment registration and test result queries, followed by online consultation. During the initial outbreak, the use of online consultation services such as team consultation, picture and text consultation, and medication consultation increased significantly. In the fairly stable period that followed, users gradually returned to making appointments and requesting test results.

**Figure 10:**
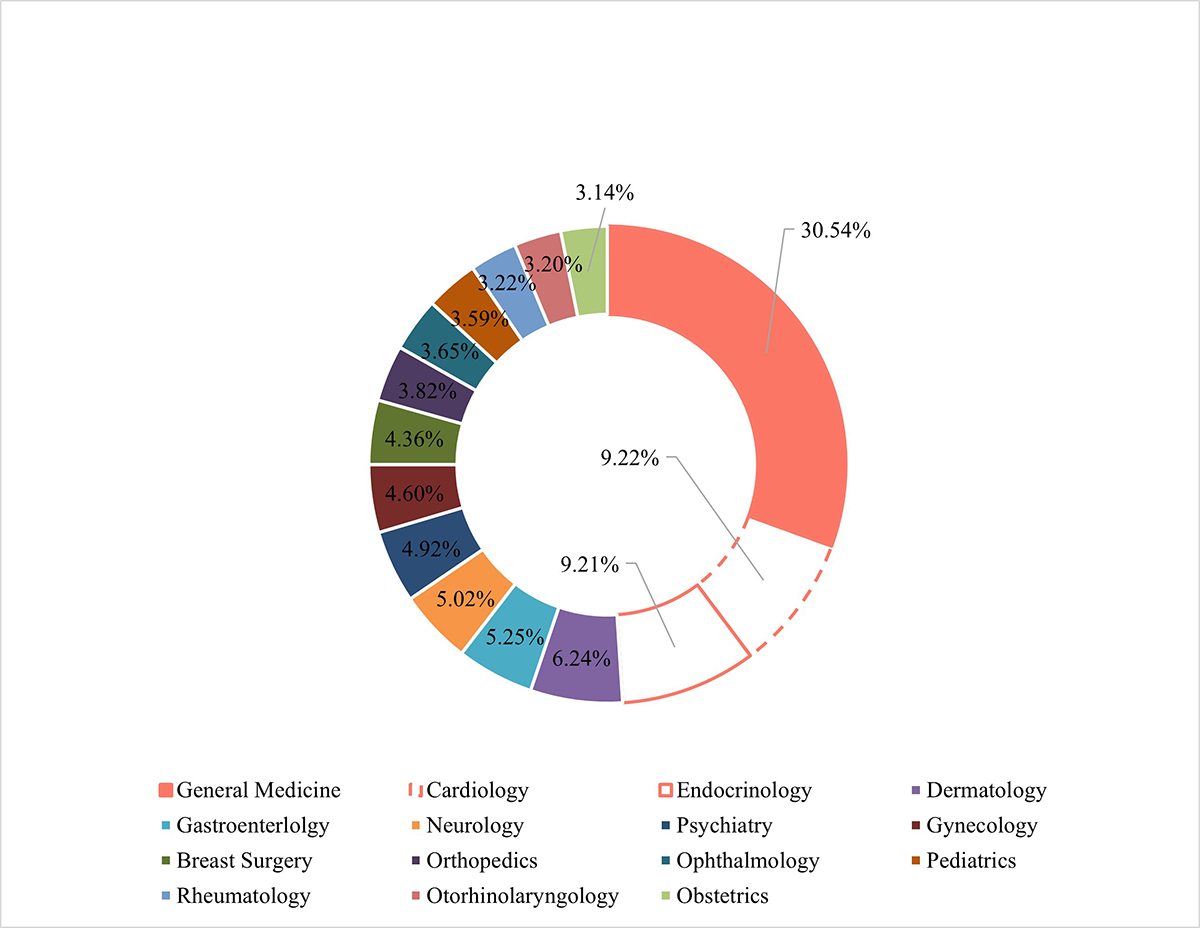
The most frequently consulted departments from January 27, 2020, to March 27, 2020.

**Figure 11:**
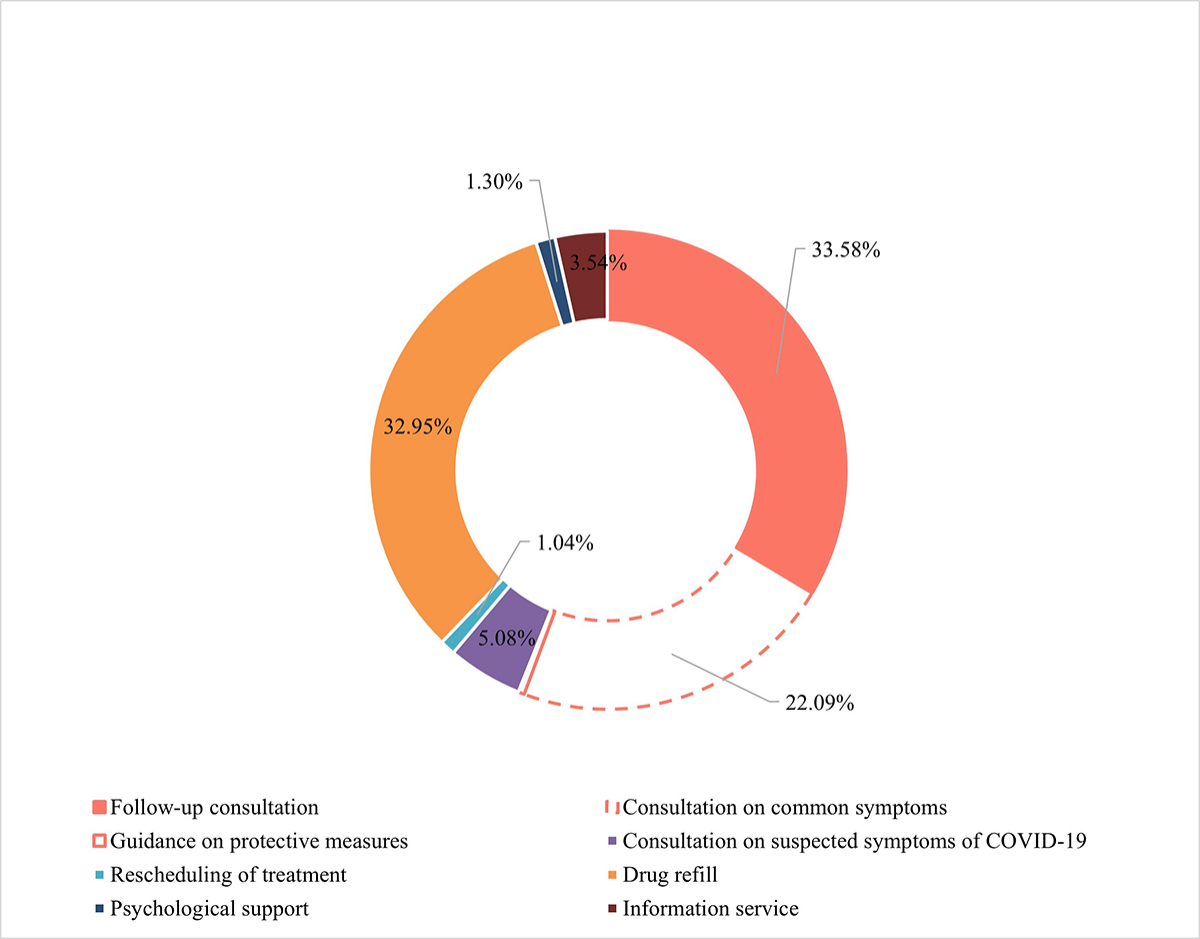
The main demands from Second Affiliated Hospital of Zhejiang University School of Medicine (SAHZU) internet hospitals patients from January 27, 2020, to March 27, 2020.

Figure 10 lists the departments that received the most consultation requests in the first outbreak period, January 27, 2020, to March 27, 2020. We identified 8 major categories of demands (Figure 11), which have shown different usage patterns over time. The distribution of the major demands was plotted in chronological order, revealing the trends in detail (Multimedia Appendix 3).

### Changes in New Registrations

**Figure 12:**
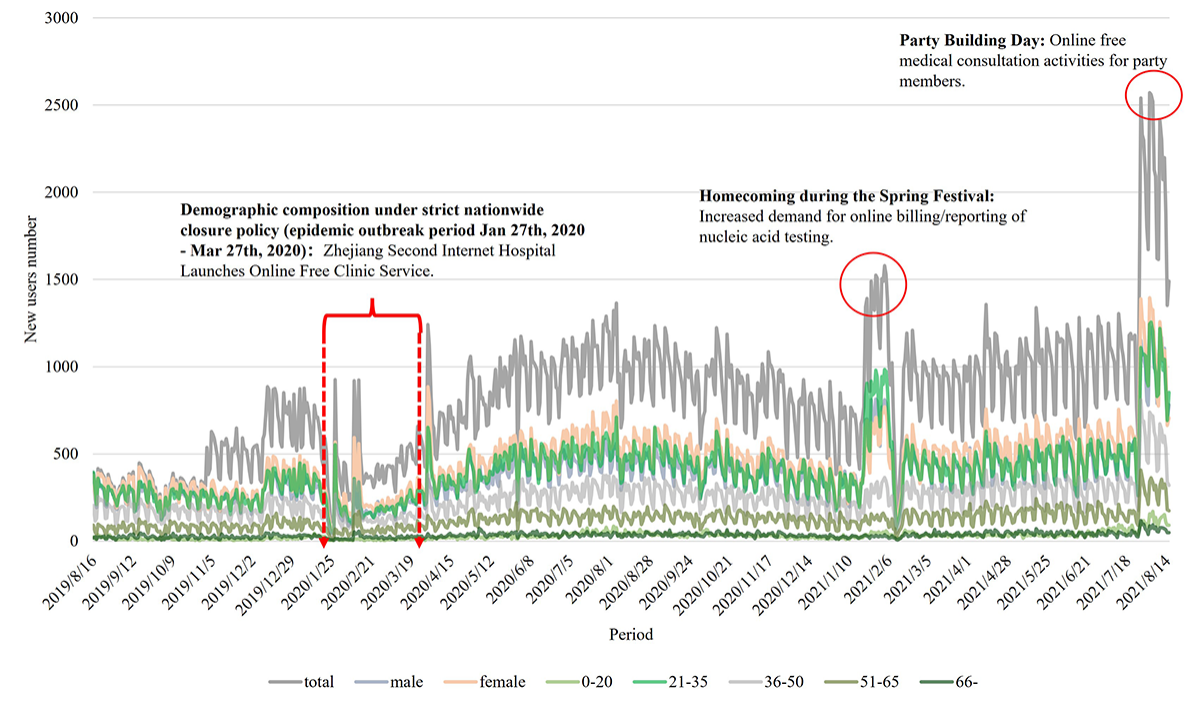
Daily Second Affiliated Hospital of Zhejiang University School of Medicine (SAHZU) internet hospital user growth from August 16, 2019, to August 15, 2021.

**Figure 13:**
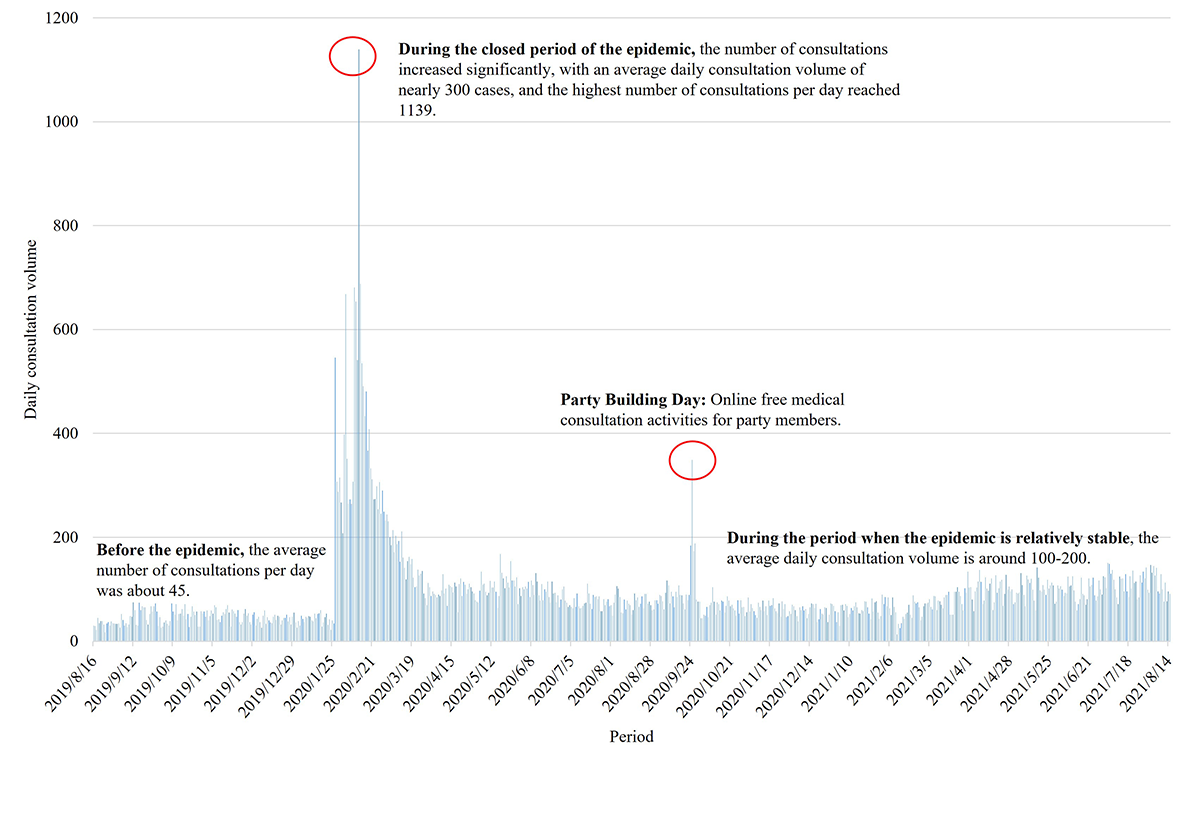
The number of new Second Affiliated Hospital of Zhejiang University School of Medicine (SAHZU) internet hospital consultations per day from August 16, 2019, to August 15, 2021.

The number of new registrations changed over time, while the main users of the SAHZU internet hospital remained 21- to 35-year-old women (Figure 12). Sudden peaks corresponded to the free consultation activities and Spring Festival. The number of consultations increased significantly during the national quarantine (Figure 13).

### ITS Analysis of New Registrations

**Figure 14:**
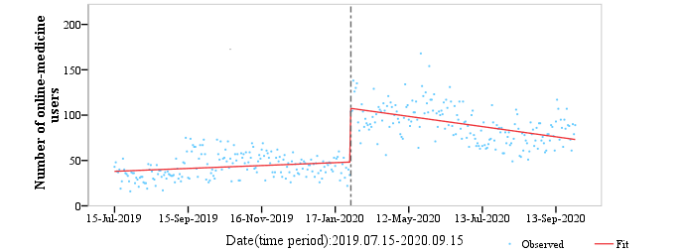
Segmented regression model for users of the online consultation platform from July 15, 2019, to September 13, 2020, using the generalized least square method; interrupted time series analysis (ITSA) to evaluate the impact of COVID-19 on telemedicine.

**Figure 15:**
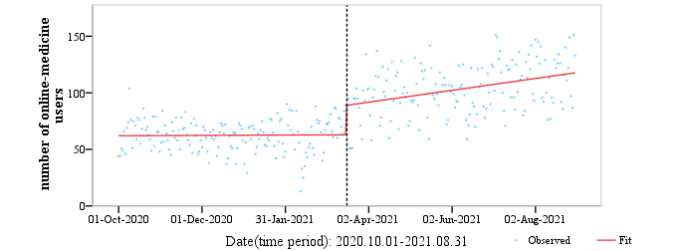
Segmented regression model for users of the online consultation platform from October 1, 2020, to August 7, 2021, using the generalized least squares method; prediction of secular trends of the prevalence of online consultations.

We conducted a 2-stage ITS analysis targeting new consultation users from July 2019 to September 2021, using data from the SAHZU internet hospital online consultation platform. The first group of data (July 15, 2019, to September 15, 2020) was used to assess the temporary effect on the utilization rate of online medical treatment attributed to domestic COVID-19 (Figure 14). The sectional point of analysis in GROUP1 was determined by the length of the lockdown in Hangzhou during the COVID-19 outbreak. Given the globalization of the COVID-19 pandemic, GROUP2 (October 1, 2020, to September 13, 2021) was used to measure long-term trends in patients' use of online medical platforms (Figure 15).

The overall model (*P*_overall_<.001) and individual coefficients (*P*_x1_=.008, *P*_x2_<.001, *P*_x3_<.001, *P*_intercept_<.001) were significant. The starting point of online medical service users was estimated at 38,162 (Table 2), with the number of active users increasing every day until January 23, 2020, according to the pre-intervention slope (n=0.052, 95% CI 0.014 to 0.091; *P*=.008). After the COVID-19 intervention was implemented (the strict quarantine policy), the number of users increased significantly (n=105.736, 95% CI 92.773 to 118.787; *P*<.001). After March 27, 2020, the development of online medical services decreased over time compared with the pre-intervention period, with a coefficient of –0.235 (95% CI –0.293 to –0.180; *P*<.001), thereby indicating a decline in telehealth users after the COVID-19 outbreak. Although the mean number of users increased, the overall development showed a downwards trend. To some extent, the pandemic had a temporary impact on the utilization rate of online medical services, although the effect on patients' habits remained to be seen.

The number of active users was initially 61,738 (Table 3). The trend in the utilization of online medical platforms was insignificant before the peak of the second wave of global COVID-19 outbreak (n=0.009, 95% CI –0.052 to 0.062; *P*=.81). COVID-19 deaths increased significantly (April 11, 2021), accounting for the change in the global situation (n=25.226, 95% CI 18.258 to 33.722; *P*<.001). The number of online users increased over time after April 9, 2020, with a coefficient of 0.167 (95% CI 0.087 to 0.247; *P*<.001). Along with Figure 15, the trend in the number of online users before and after the global pandemic peak was revealed. Before the COVID-19 pandemic, people's enthusiasm for online medical treatment had reached a plateau, and their behaviors undoubtedly began to change after the outbreak.

**Table 2:** Regression using a generalized least squares model on data from the development of the domestic pandemic from July 15, 2019, to September 15, 2020, with the pre-intervention period between July 15, 2019, and January 23, 2020; the strict quarantine period between January 23, 2020, and March 27, 2020; and the postintervention period between March 27, 2020, and September 15, 2020. Maximum lag: 1; number of observations=379; F3,379=150.3; *P*<.001.

Model	Estimation	SE	*t* value (df=379)	*P* value	95% CI
Y (intercept)	38.162	2.234	17.008	<.001	(33.611 to 42.398)
x1 (pre-intervention slope)	0.052	0.02	2.657	.008	(0.014 to 0.091)
x2 (change in intercept)	105.736	6.615	15.99	<.001	(92.773 to 118.787)
x3 (change in slope/interaction)	–0.235	0.029	–8.203	<.001	(–0.293 to 0.180)

**Table 3:** Regression to determine the long-term impact of COVID-19 on internet hospitals, using a generalized least squares model on data from the development of the domestic pandemic from October 1, 2020, to September 16, 2021, with the pre-intervention period between October 1, 2020, and April 10, 2021, and the postintervention period between April 11, 2021, and September 16, 2021. Maximum lag: 1; number of observations=331; F3,331=89.13; *P*<.001.

Model	Estimation	SE	*t* value (df=331)	*P* value	95% CI
Y (intercept)	61.738	2.796	22.194	<.001	(56.555 to 67.555)
x1 (pre-intervention slope)	0.009	0.029	0.167	.81	(–0.052 to 0.062)
x2 (change in intercept)	25.226	3.931	6.612	<.001	(18.258 to 33.722)
x3 (change in slope/Interaction)	0.167	0.041	4.104	.002	(0.087 to 0.247)

### Changes in Medical Behaviors

Integrated with the follow-up surveys, we compared the changes in SAHZU internet hospital users’ major demands, medical behaviors, and concerns, and we modelled the proportion and fluctuation of each demand through the 2D model (Table 4).

**Table 4:** Changes in medical behaviors in the 2D model.

Quadrant	Connotation	Patients’ demands: March 2020 versus June 2021	Way of accessing medical care: November 2020 versus June 2021	Users’ concerns: November 2020 versus June 2021
The first quadrant (universal type)	Specific weight, positive growth	Follow-up consultation Consultation on common symptoms Information service Others	If you feel unwell, go directly to an offline hospital, and no longer use internet hospitals. When the condition is relatively stable, the follow-ups are only conducted through the internet hospital. Due to traffic or other factors, it is hoped that most diagnosis and treatment can be carried out through internet hospitals.	Doubts about the medical safety and quality of online diagnosis and treatment
The second quadrant (improved type)	Specific weight, negative growth	Drug refill	First, consult the internet hospital for advice; then, go offline for medical service according to the doctor’s advice.	Poor timeliness of online text consultation and interaction; long wait times Higher fee Personal privacy and data security protection
The third quadrant (silent type)	Small proportion, negative growth	Rescheduling of treatment Consultation on suspected symptoms of COVID-19	Others	Others
The fourth quadrant (potential type)	Small proportion, positive growth	Psychological support Guidance on protective measures Rescheduling of treatment	N/A^a^	N/A

^a^N/A: not applicable.

## Discussion

This study conducted the first in-depth pandemic-related quantitative and qualitative analyses on changes in public behavior and the perceived factors influencing the use of internet hospitals. As a representative example of a public hospital in China, SAHZU has productively employed the practical experience of telehealth in the normalization of COVID-19 prevention and control.

### Portraits of Internet Hospital Users in China

**Figure 16:**
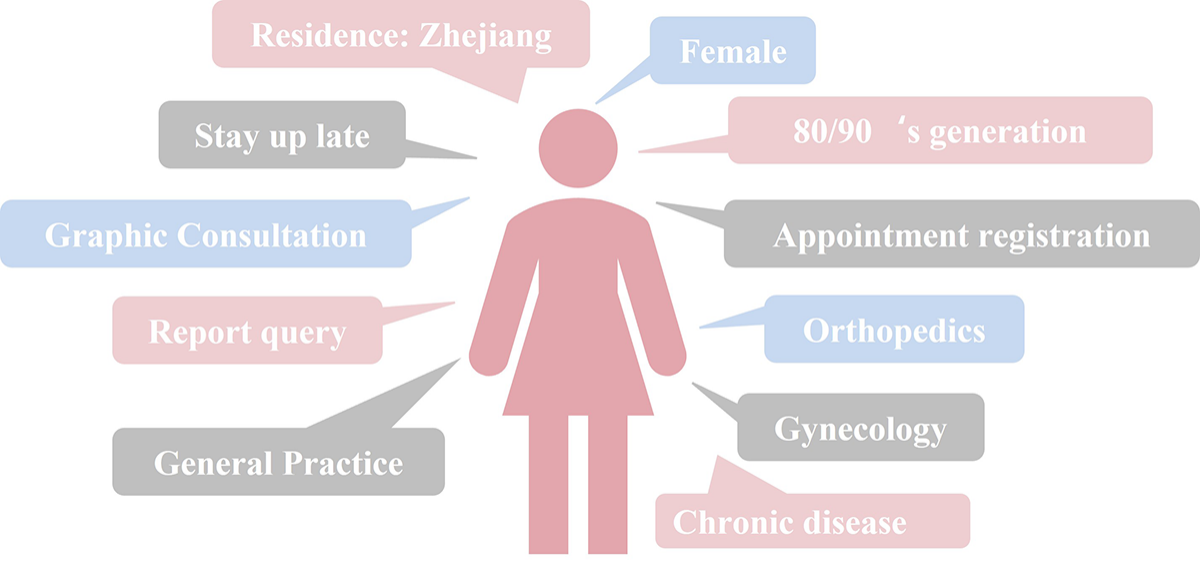
Main profiles of Second Affiliated Hospital of Zhejiang University School of Medicine (SAHZU) internet hospital users.

As a first-class public hospital and regional medical center in Zhejiang Province, the SAHZU internet hospital attracted multifarious users. The main profiles of users are shown in Figure 16.

There were slight changes in user profiles at different time points. The utilization rates of young and middle-aged women increased significantly during the pandemic isolation period. Traditional Chinese women care for the elderly and children and thus may have taken advantage of online access for their needs and those of family members.

Moreover, the popularity of the internet and improvement in digital literacy among women in China have also been reported [16,17]. Internet hospitals do not yet cover the entire population, which is reflected in the unbalanced distribution of patient profiles. Adults aged ≥66 years utilized telehealth services the least, demonstrating the greater barrier between the older population and online services. Performance risks, legal concerns, and privacy risks perceived by older adults may substantially decrease their intention to use telehealth applications [18-20].

### Acceleration of Online Medical Demands During the COVID-19 Outbreak in China

During the COVID-19 outbreak, internet access served patients and counsellors without time and space restrictions. This essential medical support reduced panic, enhanced self-protective abilities, corrected improper medical-seeking behaviors, and facilitated epidemiological screening, thereby significantly improving COVID-19 prevention and control [21,22]. The number of consultations changed with the national quarantine policy and reached a peak on February 12, 2020. The ITS analysis verified that the extreme quarantine period caused an overall increase in the number of online consultations, showing a short-term leap upwards. It turned out to be a trend to employ the internet throughout entire treatment processes. Internet hospitals enhanced patients’ sense of security, and after initial diagnosis and treatment, they could obtain online follow-ups, which further altered their medical behaviors [5].

We summarized the 8 major internet hospital user demands during the national shutdown period. In China, 60.0% of all internet hospitals provided telehealth services to address COVID-19. Internet hospitals mainly conducted consultations and psychological counselling, provided pandemic control information, filled prescriptions (86.6%), delivered drugs (74.5%), handled medical insurance claims (67.5%), and accepted or distributed donations [4]. Our results showed that follow-up consultations and drug refills were among the top requests, demonstrating efficiency and reducing unnecessary onsite visits. During the outbreak, online consultations virtually assisted in diagnosis and treatment, while surgical patients used the service for preoperative appointments and postoperative follow-up. Users with moderate health problems sought consultations more frequently than did individuals with severe conditions, indicating the value of online platforms for them, while in-person visits were essential and irreplaceable for patients with severe conditions. Additional online services, such as drug delivery, helped relieve the additional pressure on hospitals by reducing the influx of patients. In-depth efforts should be made to improve the management of prescription refills, quality and safety, dispensing acceptance, and standardization [23]. Online services offering real-time information regarding the hospital and national policies and study results indicated that users were most concerned about symptoms, up-to-date knowledge, transmission routes, and preventive measures for COVID-19 [24].

### Change in Behavior Patterns in Seeking Medical Resources

The results of this study agree with long-term data observations and surveys in different periods. During the pandemic, people's medical behavior patterns partially changed from onsite to online, which is irreversible for our public tertiary hospital. Before the pandemic, there was a temporary shock and reluctance to seek medical treatment online, but this did not produce a long-term change in the following waves of the global pandemic; people's health care habits began to change. According to the ITS analysis, this change can evolve into a long-term trend of choosing online health care and self-management. The trend in online visits followed that of overall telehealth visits, with the rates increasing dramatically after the start of the pandemic and then progressively decreasing, but users in need retained their online habits [25]. With this upwards trend, people's medical habits might have completely changed. We may carefully conclude that the response to COVID-19 will result in more than a temporary increase in online hospitals. For the predictable future, internet hospitals will reinforce the medical system by offering health care while minimizing potential exposure to the disease [26]. Once people have taken advantage of medical services via digital technologies, there is little reason to give them up.

This ongoing phenomenon should encourage health practitioners to move promptly towards digital transformation. However, how to make the ecological development of internet hospitals benign remains vague [27]. Based on the comparison of medical resource-seeking behaviors at the early and late stages of the pandemic, the changes in demands were sorted with the 2D model.

Follow-up consultation and information services were revealed as the basic needs of patients who revisited the online hospital and will be primary considerations in the future direction of internet hospital construction. Information services are capable of strengthening triage and convenience of services of internet hospitals before diagnosis.

The demand for prescriptions has dropped, indicating that some requirements cannot be resolved online, although this internet hospital function may undergo a functional iteration in the future. The limitations of internet hospitals are obvious. For instance, the demand for drug refills is significantly affected by policies (eg, the lack of health insurance or cash payments) [5]. This situation was a top-ranking medical concern regarding costs in our surveys.

Transient pandemic-related demands included consultation for common symptoms, suspected symptoms of COVID-19, psychological support, and guidance on protective measures. Online consultation serves as an indirect means of communication. Doctors consulted online are limited by the lack of information (eg, physical and auxiliary examinations) and may give only cursory medical advice, which cannot replace a hospital visit. Nondisease-specific issues and moderate health problems were much more frequent consultation requests rather than severe clinical conditions [28]. The proportion of pandemic screening was small, and although it was related to the crisis period, it may continue to rise. It is necessary to cooperate with offline approaches to more effectively draw upon the internet hospital’s online advantages.

As revealed from the analysis of the path to select medical care, as an improved demand in 2D analysis, an increasing number of patients are willing to conduct research on the internet before going offline or following a doctor's online advice afterwards. It is noteworthy that the choice of “directly choosing offline medical treatment” fluctuates to a certain extent, thereby reflecting a temporary return to traditional habits with the decline of the pandemic.

### Implications for Internet Hospital Development

Public online hospitals offer reliable resources and complete functions but have limited profit models. Long-term effective operation is an issue. Meanwhile, with novel vaccines and drugs targeting SARS-CoV-2 being developed, the challenge faced by the internet hospital community is to continuously update solutions for the majority of users. It will be crucial to consider the benefit-risk ratio for optimal therapies and minimize onsite visits. To sustain the online health care system, governments and societies are recognizing technology as a promising solution for innovative health service delivery and expansion with minimal investments.

Our surveys showed that the optimal demands of online users appear in prediagnosis and posttreatment. Human medical behaviors cannot be comprehensively shifted to digital access. Key online service implications include the entire closed loop of one’s medical behaviors, as well as refined services before and after onsite visits. In addition, the construction of a portal for specialized diseases through online forms is an important direction for future internet hospital construction.

The pandemic has given health services an impetus for managing chronic conditions in innovative ways. Notably, the departments that received the most consultations (General Medicine, Cardiology, and Endocrinology) complied with the incidence of internal medicine [29]. Patients expect online consultations to provide professional advice and personalized care. To date, personalized telehealth solutions and clear implementation recommendations are being fully explored by internet hospitals [30]. Cancer patients, as another chronic condition population, are more susceptible to infection owing to the immunosuppressed state caused by anticancer therapy. In the spectrum of high-incidence tumor diseases in China, the most common types are lung cancer (approximately 17.9% of the total new incidence), colorectal cancer (12.2%), gastric cancer (10.5%), breast cancer (9.1%), and liver cancer (9.0%) [31]. These data are echoed in the top consultation rankings of thoracic, breast, and gastroenterology departments. The online hospital acts as a vital solution for various cancer patients, along with important support for many oncologists to help with decision-making [32]. Telehealth steps are recommended for postoperative patients and those on interventions for multiple adjuvant treatments [33].

While current user profiles indicate that these are the most popular departments for online consultation, diseases with strong stigmas, such as gynecology, infertility, dermatological problems, and mental diseases, are also benefitting from telehealth solutions. Patients experiencing stigma, which refers to the inner shame of patients suffering from certain diseases who are experiencing psychological stress [34], are taking advantage of online medical resources that allow them to seek the help they need with maximum privacy, by keeping these patients at a safe distance away from virtue circumstance. For instance, telehealth has been utilized as a useful communication method in the treatment of depression, anxiety, and posttraumatic stress disorder (PTSD) during the pandemic [24,35]. By the end of January 2020, consultation rates for psychiatric issues surged. Nevertheless, online psychological support peaked during the initial lockdown weeks. In view of isolation, misinformation and rumors spread via social media. Likewise, individuals worried about contracting this unknown virus and consultation for suspected COVID-19 accounted for 5.2% of all consultations, which is consistent with parallel studies [36]. During the early outbreak of COVID-19, internet hospitals assisted in relieving psychological burdens and increased disease awareness by providing official and responsible information. Furthermore, up-to-date health information was provided to relieve social anxiety.

### Derivative Problems of Internet Hospitals

Perceived risk, defined as one’s perception of uncertainty in the use of telehealth services and the severity of its consequences, is measured with 4 constructs: privacy risk [37], performance risk, legal concern, and trust [38]. Rectifying the concerns of users for online medical behaviors is also an important issue. All the derivative problems of online medical care in the survey were sorted out, as well as the corresponding reasons and possible solutions (see Table 5). Our 2D analysis highlighted the poor timeliness of online text consultation interactions, followed by high online fees and concerns about personal privacy and data security. The potential construction direction of internet hospitals refers to how to ensure the medical safety and quality of online diagnosis and treatment. During the COVID-19 pandemic, health care professionals, designers of telehealth applications, and policy makers devised more practical functions, user-friendly interfaces, and reasonable policy guidance for internet hospitals to upgrade the existing model and to deal with future crises.

**Table 5:** Derivation problems of internet hospitals.

Problems	Corresponding reasons	Possible solutions
Form	Poor timeliness Insufficient doctor-patient interaction Unfriendly experience	Enhance information interaction reminder. Cultivate user service. Optimize the rationality of the app interface. Improve app functions, and enhance user experience.
Cost	The big price gap between internet hospitals Unable to pay with medical insurance	Issue government policies to guide prices. It is recommended that the medical insurance department include online diagnosis and treatment fees in the scope of medical insurance payment.
Ethics	Insufficient patient privacy protection technology Concerns with patient privacy leakage Lack of laws, regulations, and policy guidance	Issue policy documents to provide legal support. Clarify the identification of medical malpractice and the division of responsibility. Deidentify private data.
Platform	Incomplete and inadequate consideration of front-end, back-end, and bottom construction	Strengthen the technical team, and improve the data sharing ability and operability. Consider data security needs.

### Limitations and Improvements

The data were collected from a single institution in China. SAHZU is a public tertiary hospital and may not be representative of other levels of hospitals and different regions. However, Zhejiang University ranks the third highest among China’s universities, and the hospital works with over 200 primary and secondary hospitals. Our online hospitals cover the nation, and almost 900,000 users had registered by January 2022. Therefore, we assume, to a certain extent, that our conclusions are representative. At the same time, the application prospects of internet hospitals in primary or secondary hospitals and multicenter studies are required to validate our conclusions. The surveys we utilized enabled users to fully express their experiences without the pressure of delivering socially acceptable opinions to an interviewer. However, the actual data acquired through face-to-face interviews were more authentic. Furthermore, the consultation database might be managed by artificial intelligence (AI) and build functions such as internet AI-assisted triage.

### Conclusions

Since the outbreak of COVID-19 at the end of 2019, the pandemic has imposed great economic and social burdens worldwide. We conducted a retrospective cross-sectional study by analyzing online medical behaviors over 2 years. Our findings imply that, as a public tertiary internet hospital, the SAHZU internet hospital is partially and irreversibly integrated into the traditional medical system.

## Appendix

Multimedia Appendix 1 Questionnaire about internet hospital usage.

Multimedia Appendix 2 Interrupted time series (ITS) analysis.

Multimedia Appendix 3 Daily flow of the change in major demands for online consultation during the nationwide strict quarantine policy.

## Abbreviations

AI: artificial intelligence
GLSM: generalized least square method
ITS: interrupted time series
PTSD: posttraumatic stress disorder
SAHZU: The Second Affiliated Hospital of Zhejiang University School of Medicine

## Data Availability

The data set supporting the conclusions of this article is included within the article. Additional data are available for review upon request.
