# Supplementary material for: Experiences and Challenges of Emerging Online Health Services Combating COVID-19 in China: Retrospective, Cross-Sectional Study of Internet Hospitals
Source: JMIR Med Inform. 2022 Jun 1;10(6):e37042. doi: 10.2196/37042 (PMC9162135; doi:10.2196/37042)
Supplement: Multimedia Appendix 1 [file medinform_v10i6e37042_app1.docx]

**Multimedia Appendix 1**

**患者对互联网医院的使用意愿调查**

亲爱的先生/女士：

您好！非常感谢您抽出宝贵的时间 3-5 分钟参与本次调查。

本问卷旨在了解患者使用互联网医院的主要需求，以及在疫情前后，患者就诊方式的变化以及对使用互联网医疗服务的担忧，从而改进并优化浙二互联网医院的功能模块，提升我院的互联网医疗健康服务能力。

以下调查中获得的所有信息都是保密的，您的隐私信息不会被记录。答案不分对错，选择您认为合适的选项即可。您的配合和支持对于本研究和推动互联网医疗的应用与推广至关重要，再次感谢您的参与！

1. 您在疫情期间使用浙二互联网医院的主要需求是？（多选）

a) 向之前就诊过的医生进行复诊

b) 长期使用的药物需要进行续方

c) 出现了一些常见症状但不方便去医院

d) 怀疑自己出现了新冠相关症状

e) 需要心理疏导和支持服务

f) 咨询如何在疫情期间进行自我防护

g) 获取导医分诊、平台使用、医院何时恢复门诊和检查等信息

h) 咨询主治医生何时进行手术或检查，或修改预约时间

i) 其他

2. 疫情后，您的就诊方式最符合以下哪种情形？（单选）

a) 出现不适直接进行线下就医，不再使用互联网医院

b) 先在互联网医院上对症咨询，根据医生建议再前往线下就医

c) 病情较为稳定或变化不大时，仅通过互联网医院进行复诊

d) 由于交通或其他因素，希望能通过互联网医院进行绝大多数诊疗

e) 其他

3. 您对互联网医疗服务最大的顾虑包括？ 您对互联网医疗服务最大的顾虑包括？（多选）

a) 对网上诊疗的医疗安全和质量存在疑虑

b) 网上文字咨询互动的时效性较差，等待时间较长

c) 当前线上比线下问诊收费高

d) 对个人隐私和数据安全保护的顾虑

e) 其他

**The questionnaire of Internet hospitals usage (English version)**

Dear Sir/Madam:

Hello! Thank you very much for taking 3-5 minutes to participate in this survey.The purpose of this questionnaire is to understand the main needs of patients for using Internet hospitals, changes in their way of visits and concerns about tele-medical services, so as to optimize the functional modules of Zhejiang Second Internet Hospital and enhance the Internet medical service capacity.

All information obtained in the following investigations is confidential, and your private information will not be recorded. There is no right or wrong answer, just choose the option most appropriate in your situation. Your cooperation and support are very important for this study. Thank you again for your participation!

**1. What are your main needs for using Zhejiang Second Internet Hospital during the epidemic? (Multiple choice)**

a) Follow up with the doctor you have seen before

b) Drug refill

c) There are some common symptoms but it is not convenient to go to the hospital

d) Suspect about symptoms related to the new virus

e) Need psychological counseling and support

f) Consult how to protect yourself during the epidemic

g) Obtain information such as medical guidance and triage, platform usage, when the hospital will resume clinics and examinations, etc.

h) Consult your attending when to perform surgery or examination, or reschedule

i) Other

**2. After the epidemic, which of the following situations best suits your medical behavior? (Single choice)**

a) If you feel unwell, go directly to offline hospital, and no longer use Internet hospitals.

b) Firstly, consult on the internet hospital for advice, then go offline for medical service according to the doctor’s advice.

c) When the condition is relatively stable, the follow-ups is only conducted through the Internet hospital.

d) Due to traffic or other factors, it is hoped that most of the diagnosis and treatment can be carried out through Internet hospitals.

e) other

**3. What are your biggest concerns about Internet medical services? (Multiple choice)**

a) There are doubts about the medical safety and quality of online diagnosis and treatment.

b) The timeliness of online text consultation and interaction is poor, and the waiting time is long.

c) The current online consultation fee is higher than offline.

d) Concerns about personal privacy and data security.

e) other
