# Supplementary material for: Experiences and Challenges of Emerging Online Health Services Combating COVID-19 in China: Retrospective, Cross-Sectional Study of Internet Hospitals
Source: JMIR Med Inform. 2022 Jun 1;10(6):e37042. doi: 10.2196/37042 (PMC9162135; doi:10.2196/37042)
Supplement: Multimedia Appendix 2 [file medinform_v10i6e37042_app2.docx]

**Multimedia Appendix 2**

**Interrupted time series (ITS) analysis**

A time series of a particular outcome of interest is used to establish an underlying trend, which is ‘interrupted’ by an intervention at a known point in time, such as a disease outbreak. The data collected over time exhibited a feature in which the data points tended to be correlated (serial correlation), thereby affecting the ARIMA results. The coefficients express the underlying trend before intervention, the level variation caused by the intervention and the slope variation. A minimum of three variables are required, the basic model is expressed as follows.

$$Y_{t}=\beta_{0}+\beta_{1}*X_{1}+\beta_{2}*X_{2}+\beta_{3}*X_{1}X_{2}+\varepsilon$$

i. $X_{1}$: the time elapsed since the start of the study with the unit representing the frequency in which observations are taken (e.g., month or year);

ii. $X_{2}$: a dummy variable indicating the pre-intervention period (coded 0) or the post-intervention period (coded 1);

iii. $Y_{t}$: the outcome at time t.

Before the interrupted time series analysis, the Durbin-Waston test was performed to examine the autocorrelation of objective series. As indicated from the outcome, the serial correlation could be eliminated by applying the generalized least squares model. In the following analysis, generalized least squares were adopted to determine the linear regression model.

| Auto-correlation test | GROUP 1 | GROUP 2 |
| --- | --- | --- |
| Durbin-Waston test (Linear-regression) | 1.264 | 1.456 |
| Durbin-Waston test (Prais-Winsten) | 2.027 | 1.953 |

The intervention of GROUP1 (2019/07/15-2020/09/15) can be defined as the outbreak of the domestic epidemic. The turning point of the second global COVID-19 pandemic is seen as the implementation of intervention in GROUP2 (2020/10/01-2021/09/13). If the value of the Durbin-Waston test is close to 2, there is no serial correlation.
