# Supplementary material for: Experiences and Challenges of Emerging Online Health Services Combating COVID-19 in China: Retrospective, Cross-Sectional Study of Internet Hospitals
Source: JMIR Med Inform. 2022 Jun 1;10(6):e37042. doi: 10.2196/37042 (PMC9162135; doi:10.2196/37042)

**Multimedia Appendix 3**

**Daily flow of major demands change in online consultation**

**during the nationwide strict quarantine policy**

The major demands during Jan 27^th^ to Mar 27^th^, 2020 have shown different patterns over time. The distribution of the major demands was plotted in the chronological order, and the trends they appeared were demonstrated below in details.

**
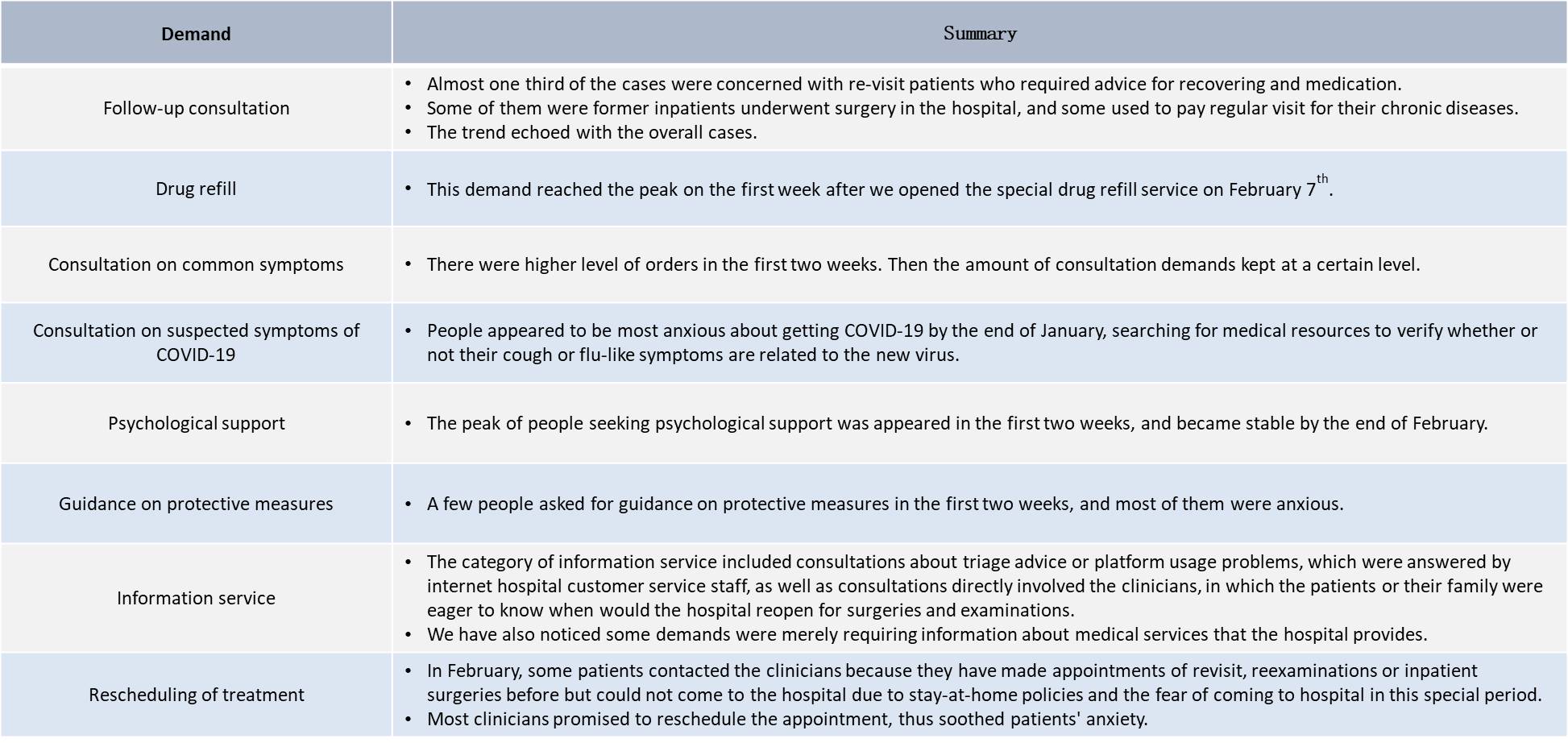
**

**Daily flow of major demands**


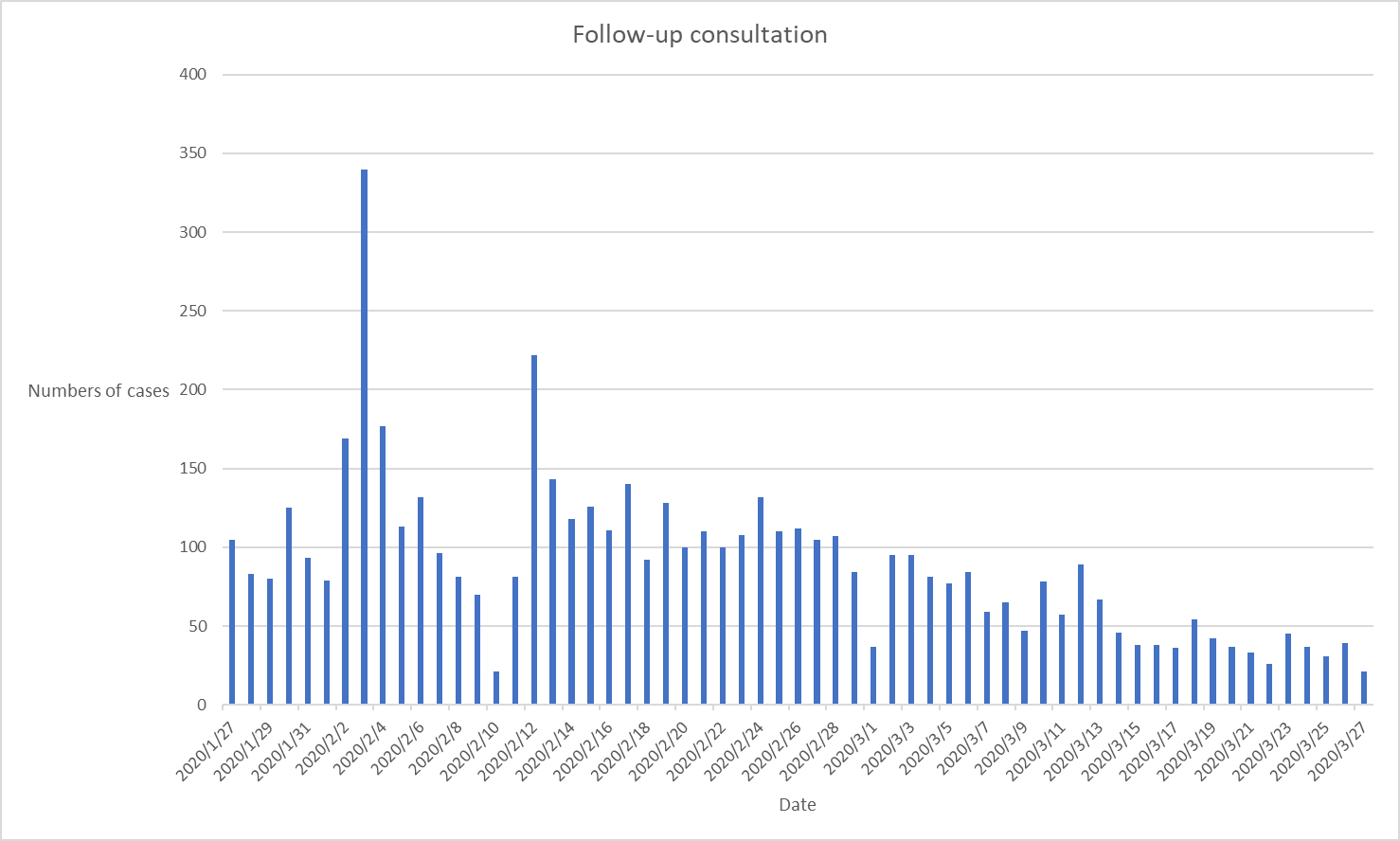


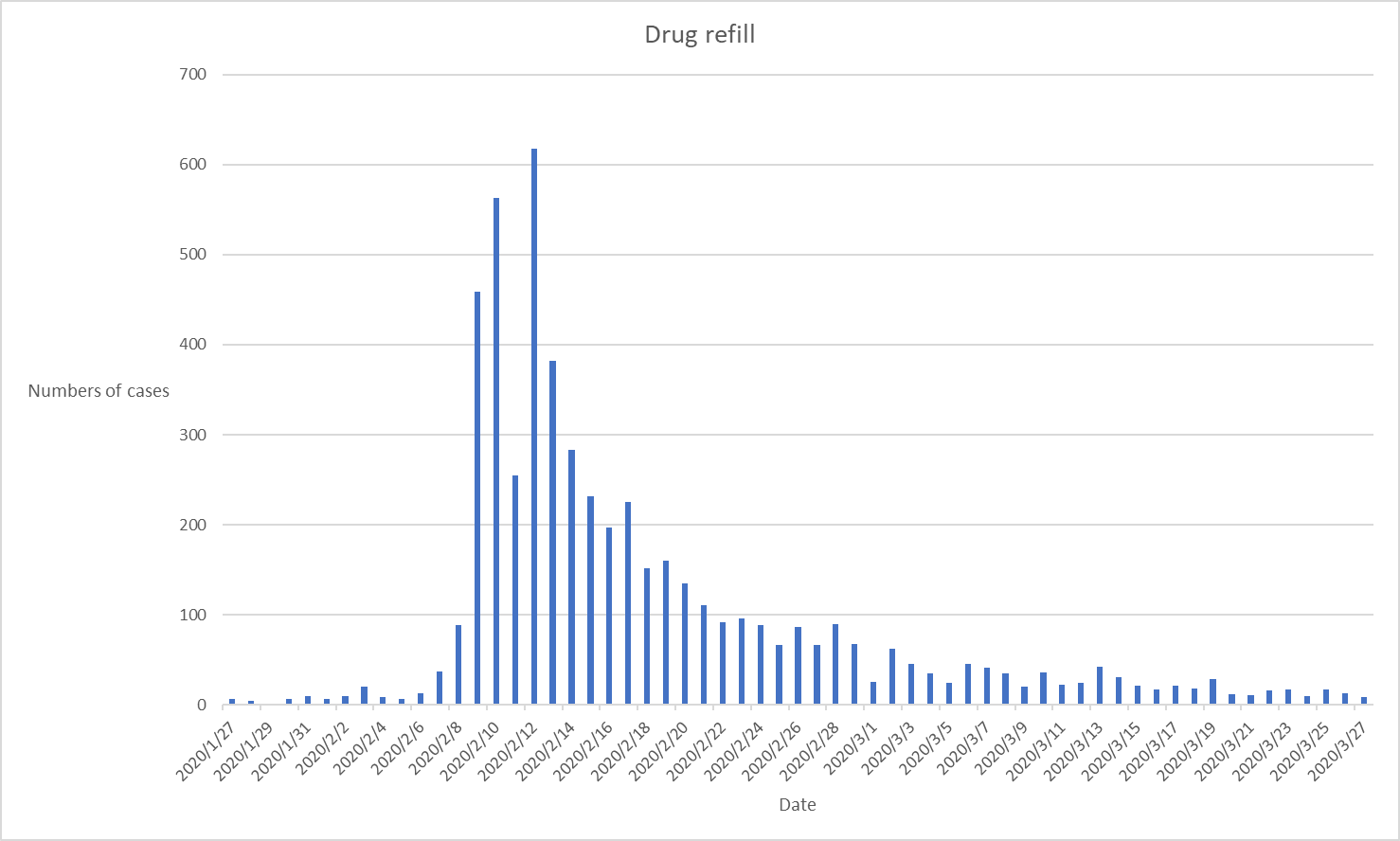


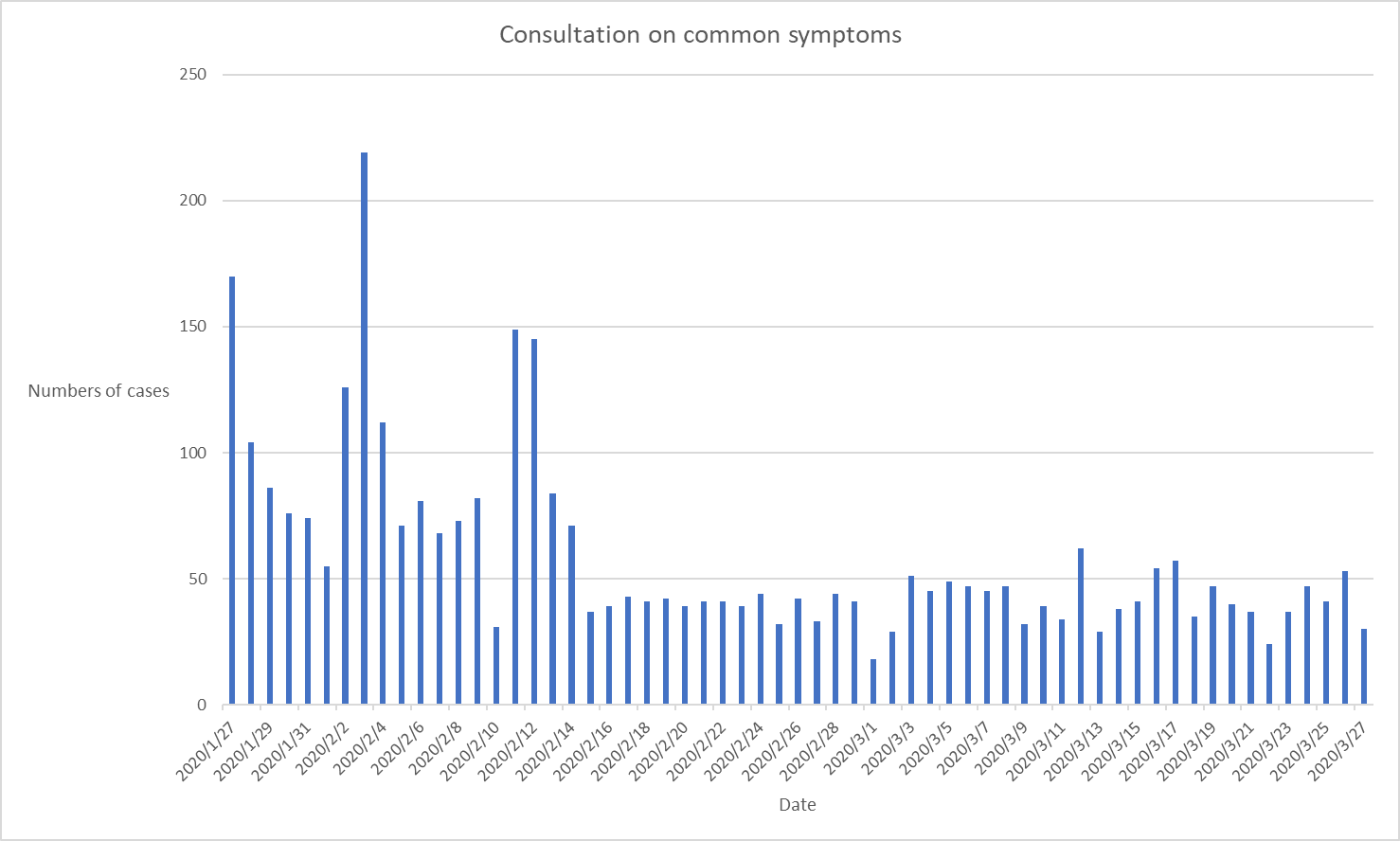


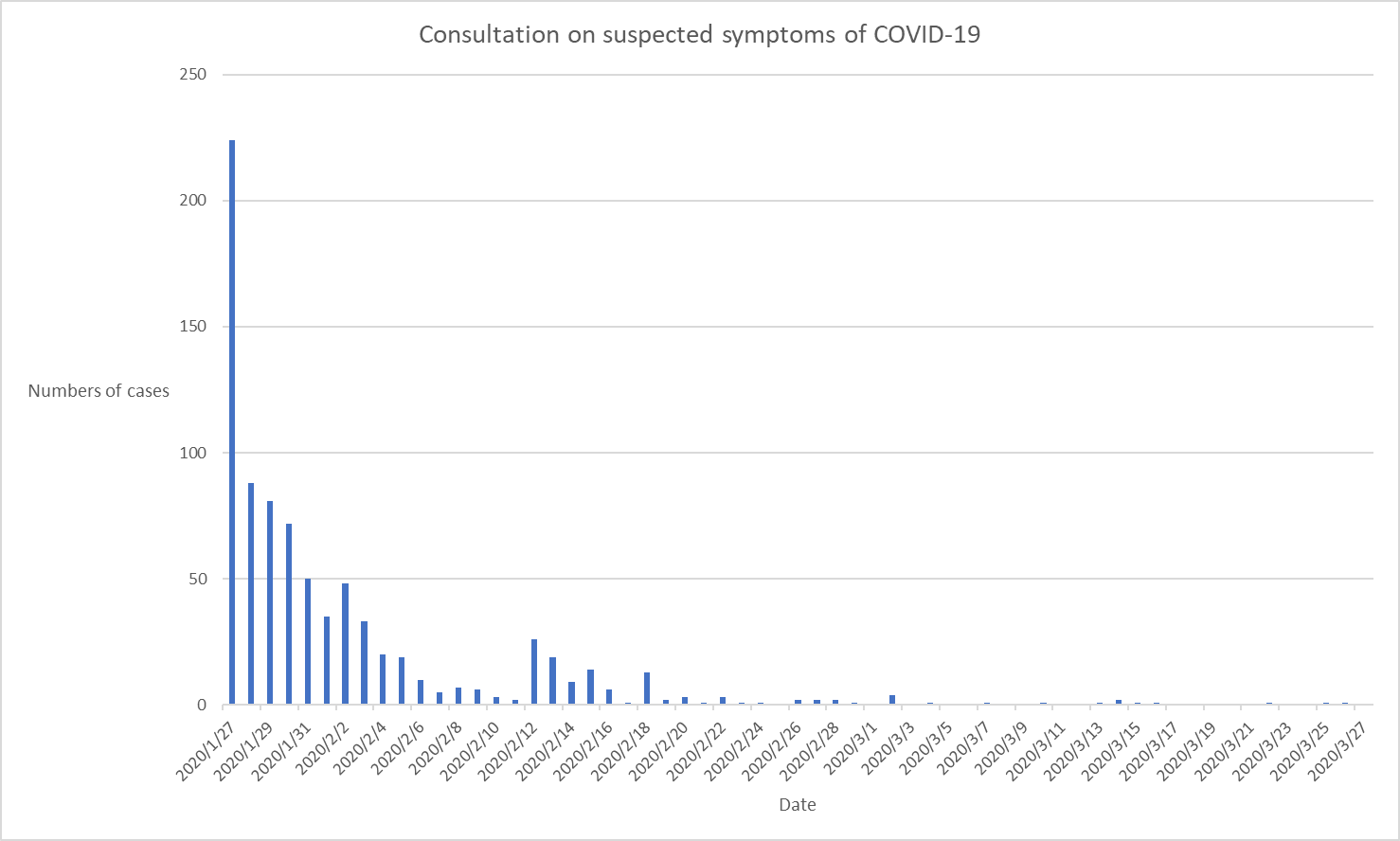


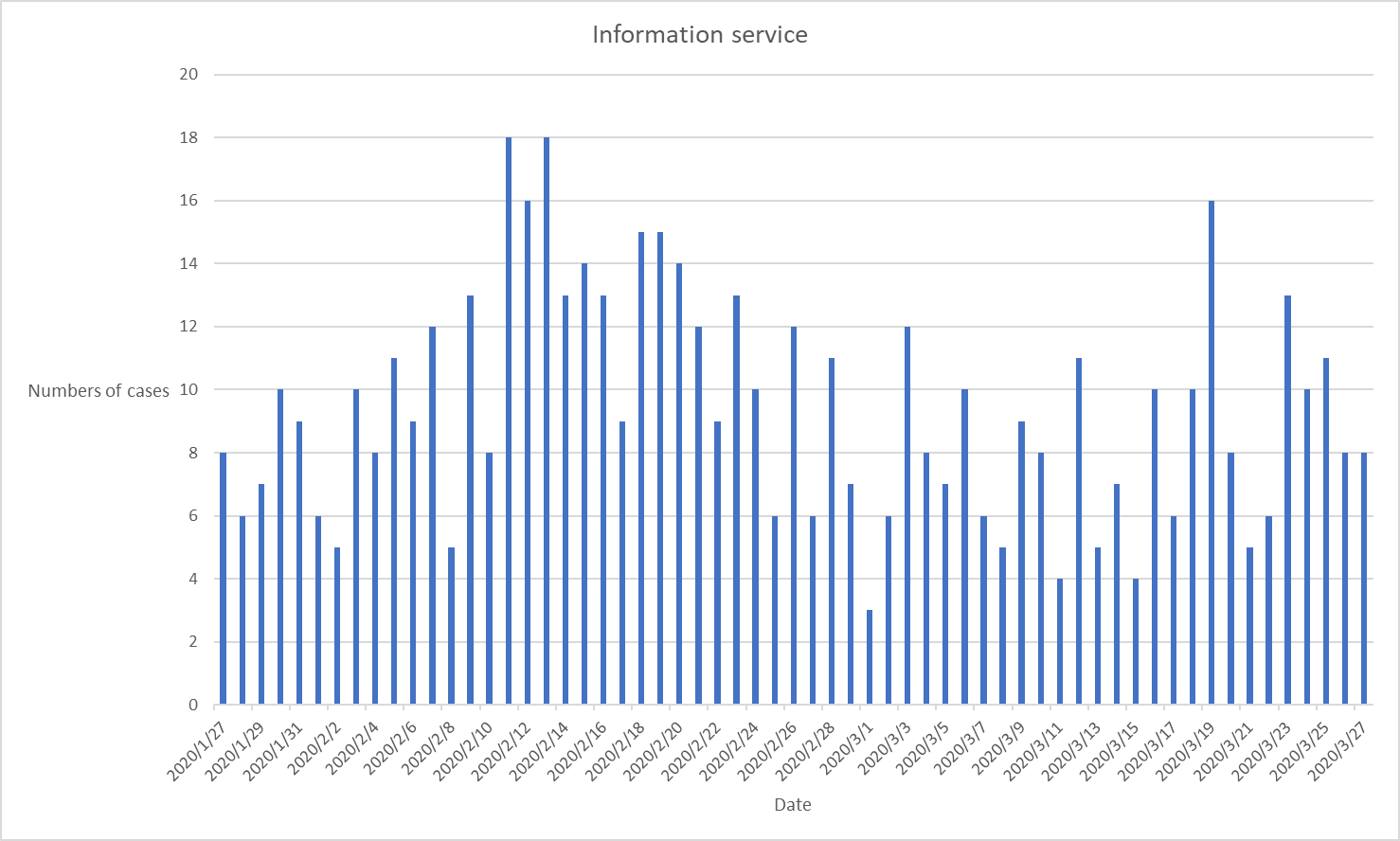


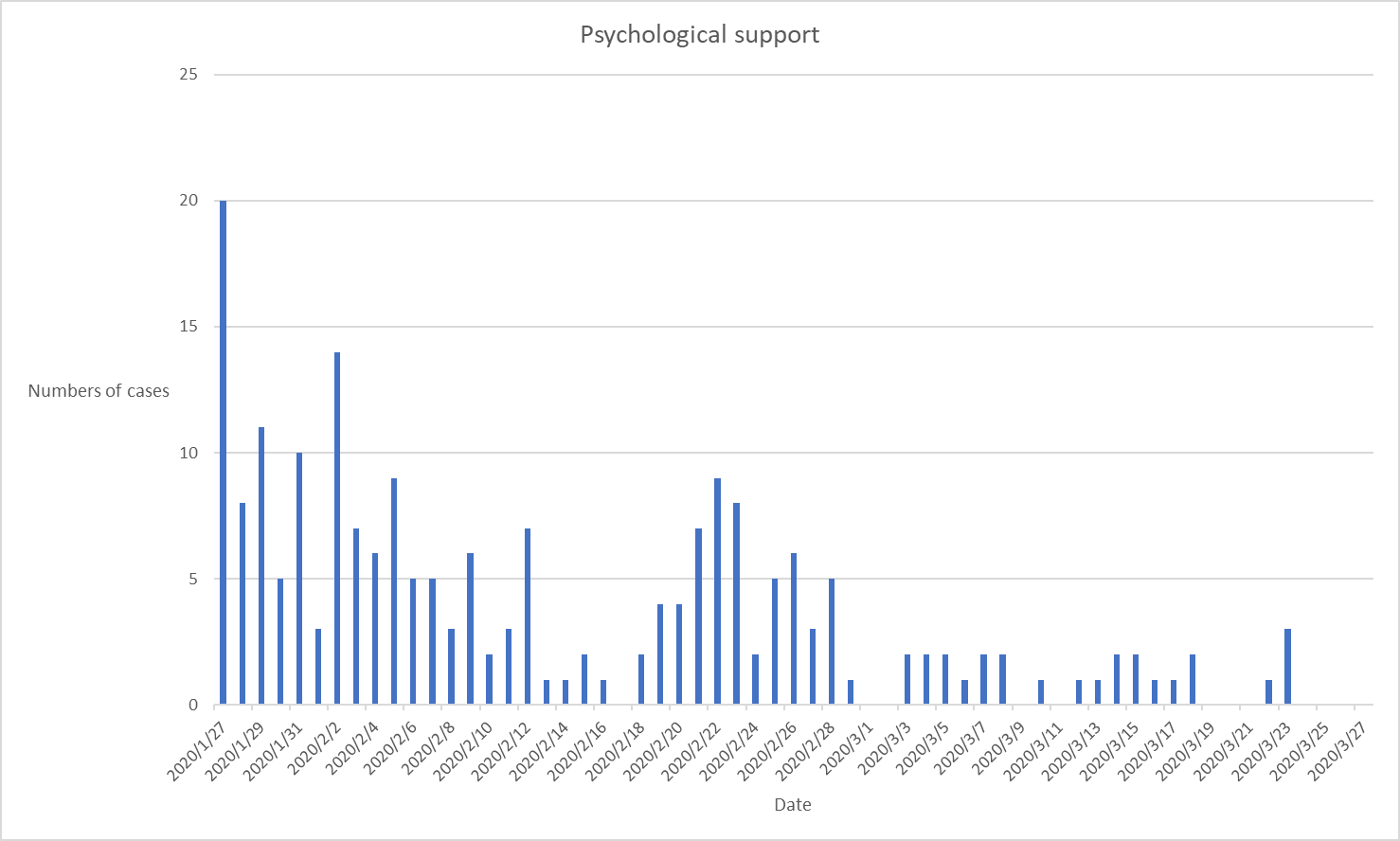


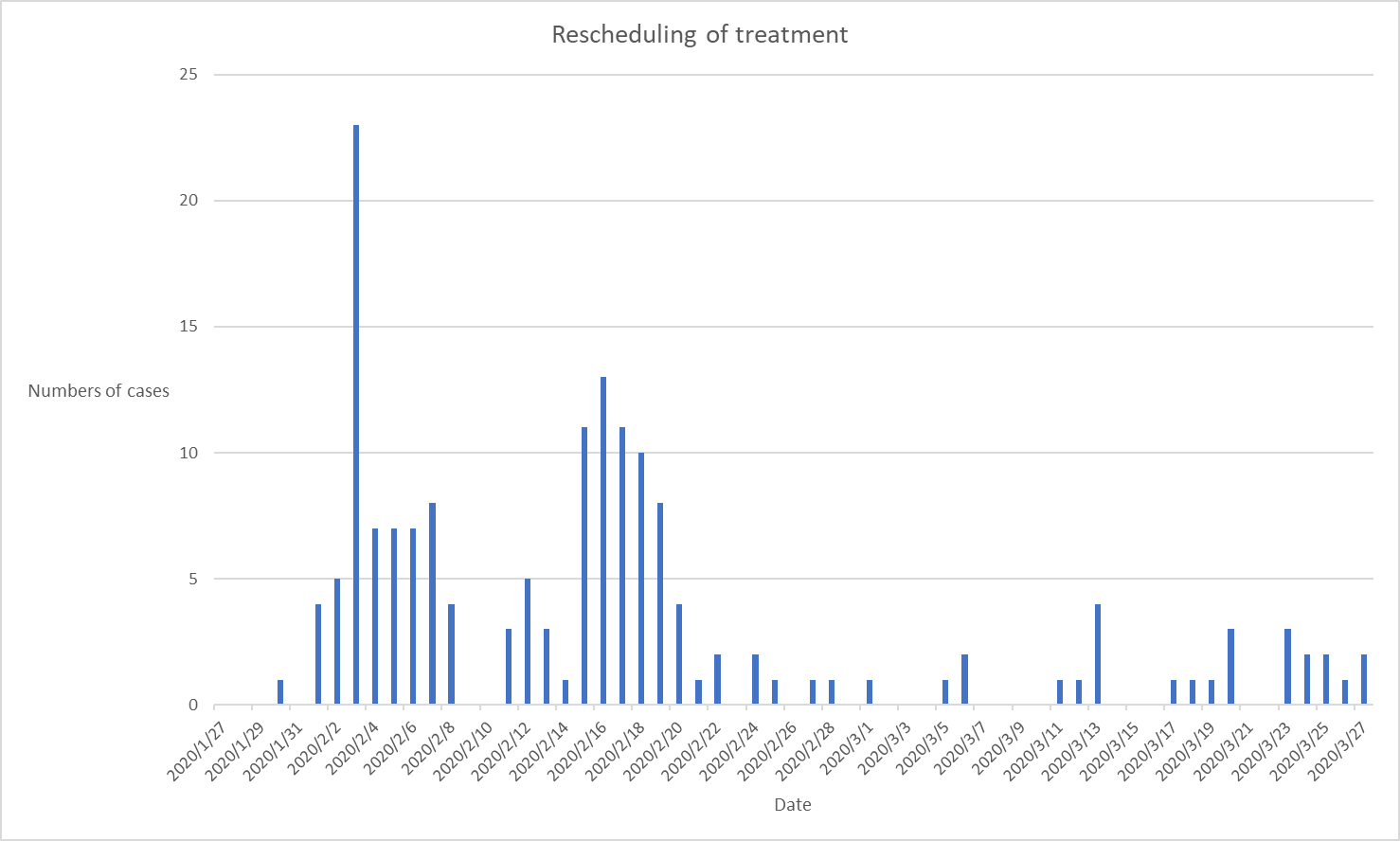


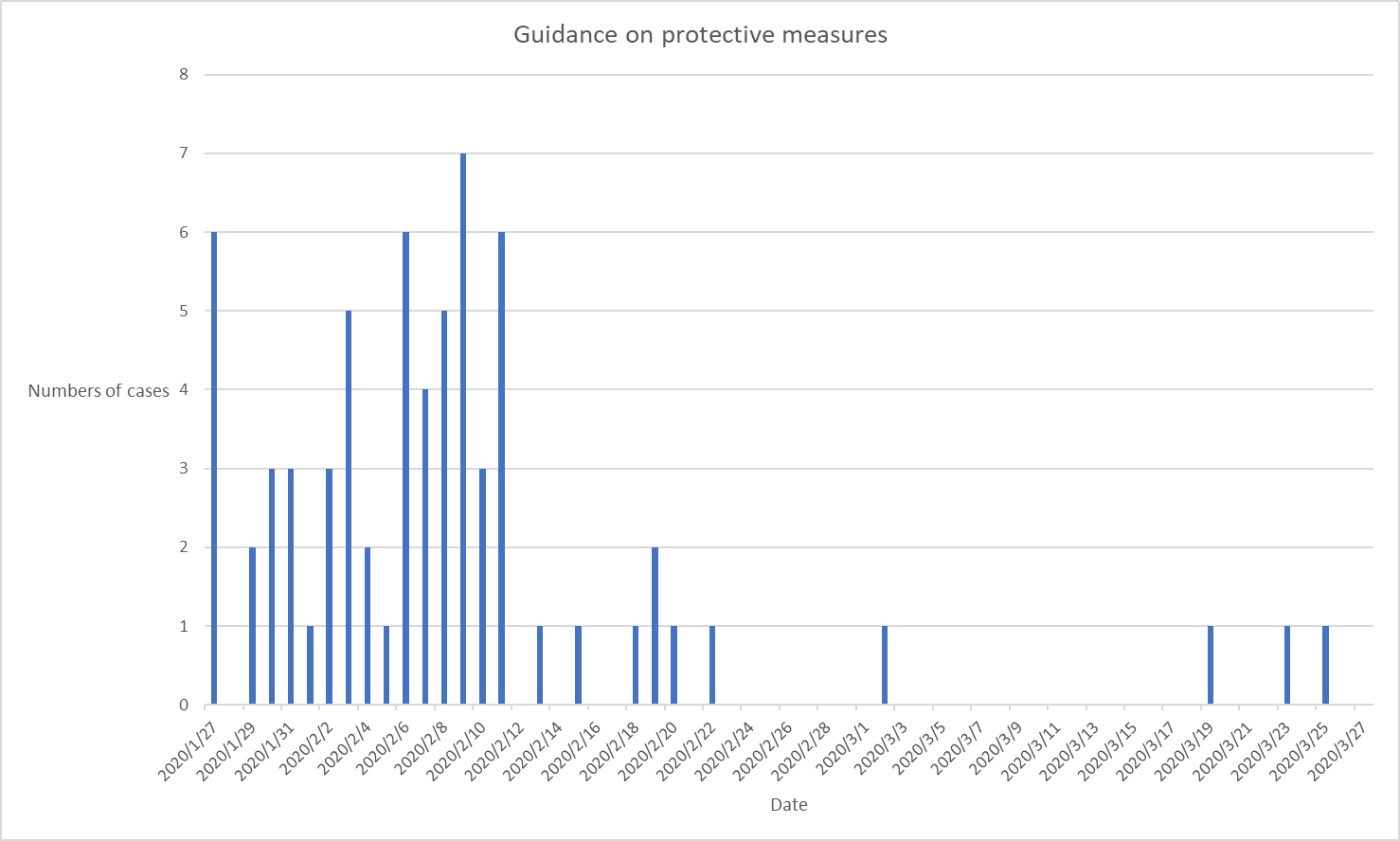

Supplement: Multimedia Appendix 3 [file medinform_v10i6e37042_app3.docx]
